# The causal relationship between severe mental illness and risk of lung carcinoma

**DOI:** 10.1097/MD.0000000000037355

**Published:** 2024-03-15

**Authors:** Xiaohan Chen, Shudan Wang, Weiyu Shen

**Affiliations:** a The Affiliated Lihuili Hospital, Ningbo University, Ningbo, China.

**Keywords:** lung carcinoma, mendelian randomization, schizophrenia, severe mental illness, smoking

## Abstract

Observational studies have suggested a link between severe mental illness (SMI) and risk of lung carcinoma (LC); however, causality has not been established. In this study, we conducted a two-sample, two-step Mendelian randomization (MR) investigation to uncover the etiological influence of SMI on LC risk and quantify the mediating effects of known modifiable risk factors. We obtained summary-level datasets for schizophrenia, major depressive disorder (MDD), and bipolar disorder (BD) from the Psychiatric Genomics Consortium (PGC). Data on single nucleotide polymorphisms (SNPs) associated with lung carcinoma (LC) were sourced from a recent large meta-analysis by McKay et al. We employed two-sample MR and two-step MR utilizing the inverse variance weighted method for causal estimation. Sensitivity tests were conducted to validate causal relationships. In two-sample MR, we identified schizophrenia as a risk factor for LC (OR = 1.06, 95% CI 1.02–1.11, *P* = 3.48E-03), while MDD (OR = 1.18, 95% CI 0.98–1.42, *P* = .07) and BD (OR = 1.07, 95% CI 0.99–1.15, *P* = .09) showed no significant association with LC. In the two-step MR, smoking accounted for 24.66% of the schizophrenia-LC risk association, and alcohol consumption explained 7.59% of the effect. Schizophrenia is a risk factor for lung carcinoma, and smoking and alcohol consumption are the mediating factors in this causal relationship. LC screening should be emphasized in individuals with schizophrenia, particularly in those who smoke and consume alcohol regularly.

## 1. Introduction

Lung carcinoma (LC) is among the most prevalent cancers globally, accounting for approximately 2.2 million new cases and 1.79 million deaths each year. It is a primary contributor to cancer-related mortality worldwide.^[1]^ Therefore, early screening and diagnosis of lung carcinoma is imperative. Identifying high-risk populations, particularly smokers, and implementing advanced early screening measures can substantially improve diagnostic rates and reduce mortality.^[2]^

Severe mental illnesses (SMI), including schizophrenia, major depressive disorder (MDD), and bipolar disorder (BD), are a global concern, with a growing number of affected individuals.^[3]^ Previous studies have shown that patients with SMI experience a higher mortality rate related to lung cancer,^[4]^ even after adjusting for factors such as age, year of cancer diagnosis, and cancer type.^[5]^ However, they receive fewer lung cancer screenings than the general population.^[6]^ Therefore, it is crucial to investigate the association between SMI and the incidence of lung cancer. This understanding can raise awareness among both patients and healthcare institutions, potentially leading to improved early screening for lung cancer, ultimately benefiting patients. While many studies have reported an elevated lung cancer risk in patients with SMI,^[7,8]^ a study has produced conflicting results.^[9]^ Moreover, previous research may be susceptible to confounding factors due to variations in diagnostic criteria, limited sample sizes, and heterogeneity in the meta-analyses. Additionally, observational studies are subjected to confounding variables such as smoking,^[10]^ alcohol consumption,^[11]^ physical activity,^[12]^ obesity,^[13]^ diabetes,^[14]^ and the influence of antipsychotic medications on SMI treatment.^[15]^ Therefore, employing approaches that provide accurate and robust estimates of exposure-outcome causality are essential for elucidating the relationship between SMI and lung cancer incidence.

Mendelian randomization (MR) is a methodology that utilizes genetic variants as instrumental variables (IVs) to estimate causal relationships free from the influence of confounding factors and reverse causality.^[16]^ It complements traditional epidemiological approaches by capitalizing on Mendel’s Second Law, which entails the random allocation of alleles during gamete formation, leading to randomized assignment of exposures.^[17]^ This allocation typically occurs independent of environmental risk factors and precedes the onset of diseases and related risk factors. Two-step MR, a variant of MR, investigates the potential mediator roles between exposure and outcomes using a two-sample MR strategy.

In this study, a two-sample MR analysis was performed to estimate the association between SMI, including schizophrenia, MDD, and BD, and the risk of lung carcinoma. Additionally, we employed a two-step MR approach to explore the mediating effects of smoking initiation, alcohol consumption, physical activity, body mass index (BMI), and type 2 diabetes on the causal link between SMI and lung carcinoma.

## 2. Materials and methods

### 2.1. Study design

**Figure 1.**
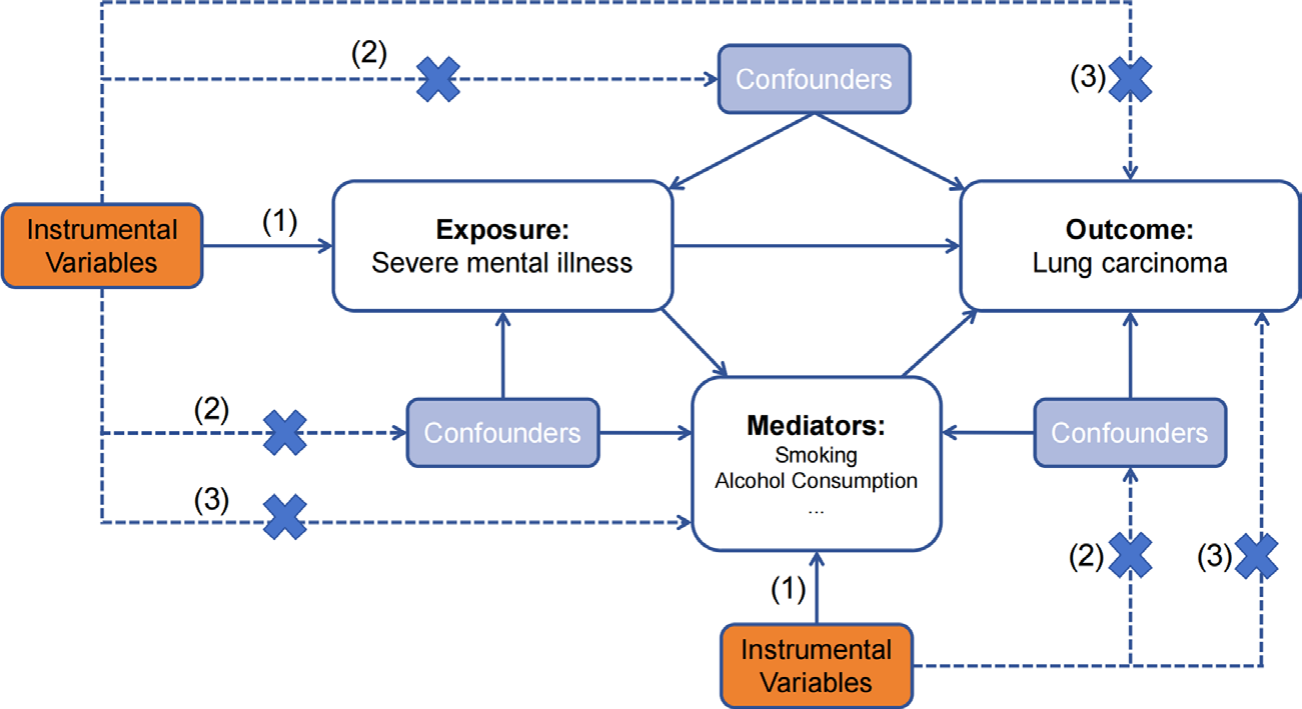
The flowchart of the study. The flowchart of the study based on three assumptions: (1) the IVs must be significantly connected to the exposure; (2) the IVs cannot be connected to any known confounders that could alter the association between an exposure and an outcome; and (3) the IVs must be unrelated to the outcome and may only affect the outcome through their effects on the exposure. This Figure illustrates a diagram of our MR study. The dash lines indicate irrelevance, and the solid lines indicate relevance. IV = instrumental variable.

This was a Mendelian Randomization (MR) study, encompassing both a two-sample MR and a two-step MR (Fig. 1). We rigorously adhere to 3 fundamental MR assumptions: strong correlations exist between IVs and exposure (association); IVs are independent of confounders (independence); and the effect of IVs on outcomes is solely mediated through exposure (exclusion restriction criteria).

All the datasets utilized in this study were publicly accessible. Ethical permission and written informed consent were obtained from the original studies.

### 2.2. Sources of genetic data

#### 2.2.1. Genetic data for exposures.

Genome-wide association study (GWAS) data for schizophrenia, major depression disorder (MDD), and bipolar disorder (BD) are available from the Psychiatric Genomics Consortium (PGC) (https://www.med.unc.edu/pgc/; Table 1). PGC stands out as one of the largest, pioneering, and highly productive consortia in the field of psychiatric research.

**Table 1:** Genetic instrumental variables exposures and outcome.

Phenotype	Data source	Sample size	N case	N control	Population
Lung carcinoma	McKay JD’s Study	85,716	29,266	56,450	European
Schizophrenia	PGC	130,644	53,386	77,258	European
Major depression	PGC	807,553	246,363	561,190	European, NR
Bipolar disorder	PGC	51,710	20,352	31,358	European

The single nucleotide polymorphism (SNPs) linked to schizophrenia were derived from a meta GWAS study^[18]^ that encompassed 90 cohorts of European (EUR) and East Asian (ASN) ancestry, along with nine cohorts of African-American (AA) and Latino (LAT) ancestry. For our analysis, we specifically utilized genome-wide association data pertaining to individuals of European ancestry comprising 53,386 cases and 77,258 controls.

We employed the most significant GWAS meta-analysis^[19]^ to identify MDD-related SNPs. This comprehensive GWAS incorporated data from three large-scale depression studies,^[20–22]^ involving a total of 246,363 European ancestry cases and 561,190 European ancestry controls after the removal of overlapping samples.

We utilized summary data for BD-related SNPs from a substantial GWAS meta-analysis^[23]^ that encompassed 20,352 cases and 31,358 controls of European descent.

#### 2.2.2. Genetic data for potential mediators.

The SNPs associated with mediators were sourced from the online GWAS summary database (https://gwas.mrcieu.ac.uk/), which encompasses smoking initiation, alcohol consumption, physical activity, body mass index (BMI), and type 2 diabetes (Table S1, Supplemental Digital Content, http://links.lww.com/MD/L872).

SNPs related to smoking initiation and alcohol consumption were selected from the GSCAN (GWAS and Sequencing Consortium of Alcohol and Nicotine use), a global consortium conducting genetic correlation meta-analyses.^[24]^ For physical activity-related SNPs, data were sourced from the UK Biobank, which conducted the largest GWAS of physical activity using a combination of self-report measures and wrist-worn accelerometer data.^[25]^ BMI and type 2 diabetes-related SNPs were obtained from the MRC Integrative Epidemiology Unit, whose GWAS ID is ukb-b-19953 and ukb-b-13806, respectively.

#### 2.2.3. Genetic data for outcome.

Summary statistics for lung carcinoma (LC) were obtained from a recent large meta-analysis.^[26]^ This meta-analysis included 14,803 cases and 12,262 controls of European descent who were genotyped using OncoArray. These data were then combined with existing data from a previous lung cancer GWAS, comprising 14,436 cases and 44,188 control samples.^[27–29]^ There was no overlap between previous GWAS and the current dataset. We performed MR analysis using summary statistics of total lung carcinoma, with 29,266 cases and 56,450 controls (Table 1).

### 2.3. Screening for instrumental variables

We identified valid IVs through the following steps. Initially, we selected SNPs with *P* < 5 × 10^−8^ from the source GWAS (relevance assumption). Subsequently, we conducted a clumping process (*r*^2^ < 0.001, clumping window = 10,000 kb)^[30]^ with reference to the 1000 Genomes European Panel.^[31]^ Additionally, we calculated F-statistics for the selected SNPs,^[32,33]^ and SNPs with an F-statistic below 10 were excluded.^[30]^ We then excluded SNPs that exhibited a significant relationship (*P* < 5 × 10^−8^) with the outcome (third assumption). Next, we used PhenoScanner V2 to exclude SNP associated with potential confounders.^[34,35]^ Ultimately, 197 SNPs were selected as instrumental variables (Table S2, Supplemental Digital Content, http://links.lww.com/MD/L873).

### 2.4. MR analyses

In the Two-Sample Mendelian Randomization (MR) analysis, the primary statistical model used was the inverse-variance weighting (IVW) approach.^[36]^ The fixed IVW model was employed when all instrumental variables (IVs) were considered valid and exhibited no pleiotropic effects, as recommended.^[37]^ In cases where there was high heterogeneity among single nucleotide polymorphisms (SNPs), the random-effects IVW model was utilized.^[38]^ A set of complementary analyses was performed, including the weighted median method,^[39]^ MR-Egger,^[40]^ and MR Pleiotropy Residual Sum and Outlier (MR-PRESSO).^[41]^

Given the elevated prevalence of smoking, alcohol consumption, overweight and obesity, physical inactivity, and type 2 diabetes among individuals with SMI, we conducted a two-step MR study. Initially, we calculated the causal effect of exposure (SMI) on the mediator variable (beta1). In the second step, we used instrumental variables (IVs) significantly associated with the mediators to assess the causal link between the mediators and the risk of the outcome (lung carcinoma) (beta2). We define beta0 as the causal effect between the exposure and outcome. If beta0, beta1, and beta2 are all statistically significant, we can conclude that there is a causal relationship between exposure and outcome, and the mediator variables partially mediate this relationship. In essence, beta1 × beta2 represents the mediating effect of exposure to the outcome, and the proportion of this mediating effect within the causal relationship is calculated as beta1 × beta2/beta0.^[42]^ However, if beta0 was significant while neither beta1 nor beta2 were significant, it would suggest that the mediator does not play a mediating role in the causal association from exposure to the outcome.

### 2.5. Sensitivity analysis

To ensure the reliability of our results, we conducted sensitivity analyses to detect potential horizontal pleiotropy and heterogeneity. The IVW method and Cochran’s Q statistic of the MR-Egger test were used to detect heterogeneity. We defined *P* > .05 as no heterogeneity among IVs.

For pleiotropy testing, we employed the intercept test in the MR-Egger analysis.^[43]^ This analysis allowed us to calculate causal estimates after correcting for pleiotropic effects.^[40]^ We excluded the influence of pleiotropy at the IV level when the intercept approached zero (<0.1) and the *P* value exceeded .05. Additionally, we employed the MR-PRESSO method to identify pleiotropic outliers and generates unbiased estimates after excluding outlier SNPs.^[41]^ If outlier SNPs were detected, we excluded these SNPs and re-ran other MR analysis approaches. We also conducted a leave-one-out analysis to assess whether causality was dependent on or biased by a single SNP.

All data in this study were analyzed using TwoSampleMR ver. 0.5.6 and R ver. 4.1.3. Statistical significance was set at *P* < .05.

## 3. Results

### 3.1. Schizophrenia–LC risk association

**Figure 2.**
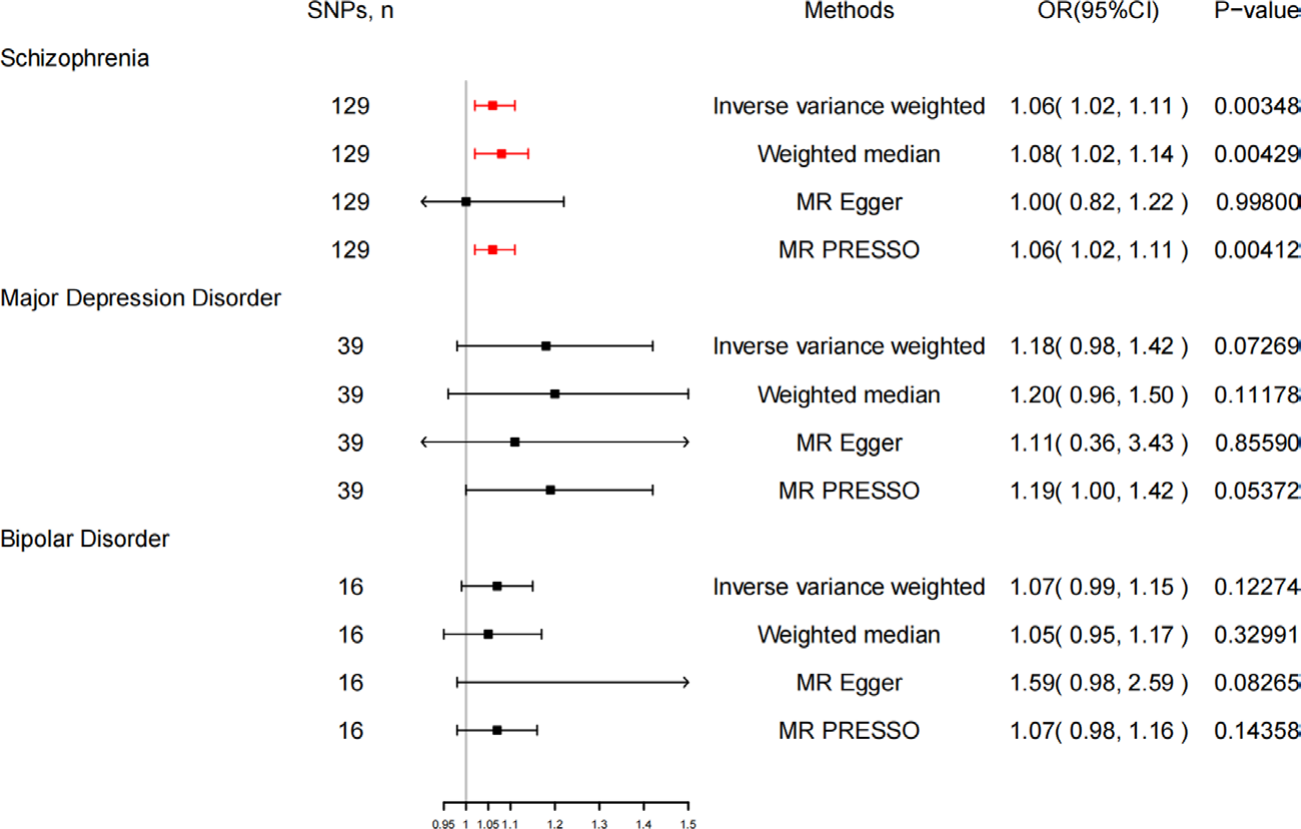
Associations of schizophrenia, major depressive disorder (MDD), and bipolar disorder (BD) with lung carcinoma. CI = confidence interval, OR = odds ratio, SNP = single nucleotide polymorphism.

**Figure 3.**
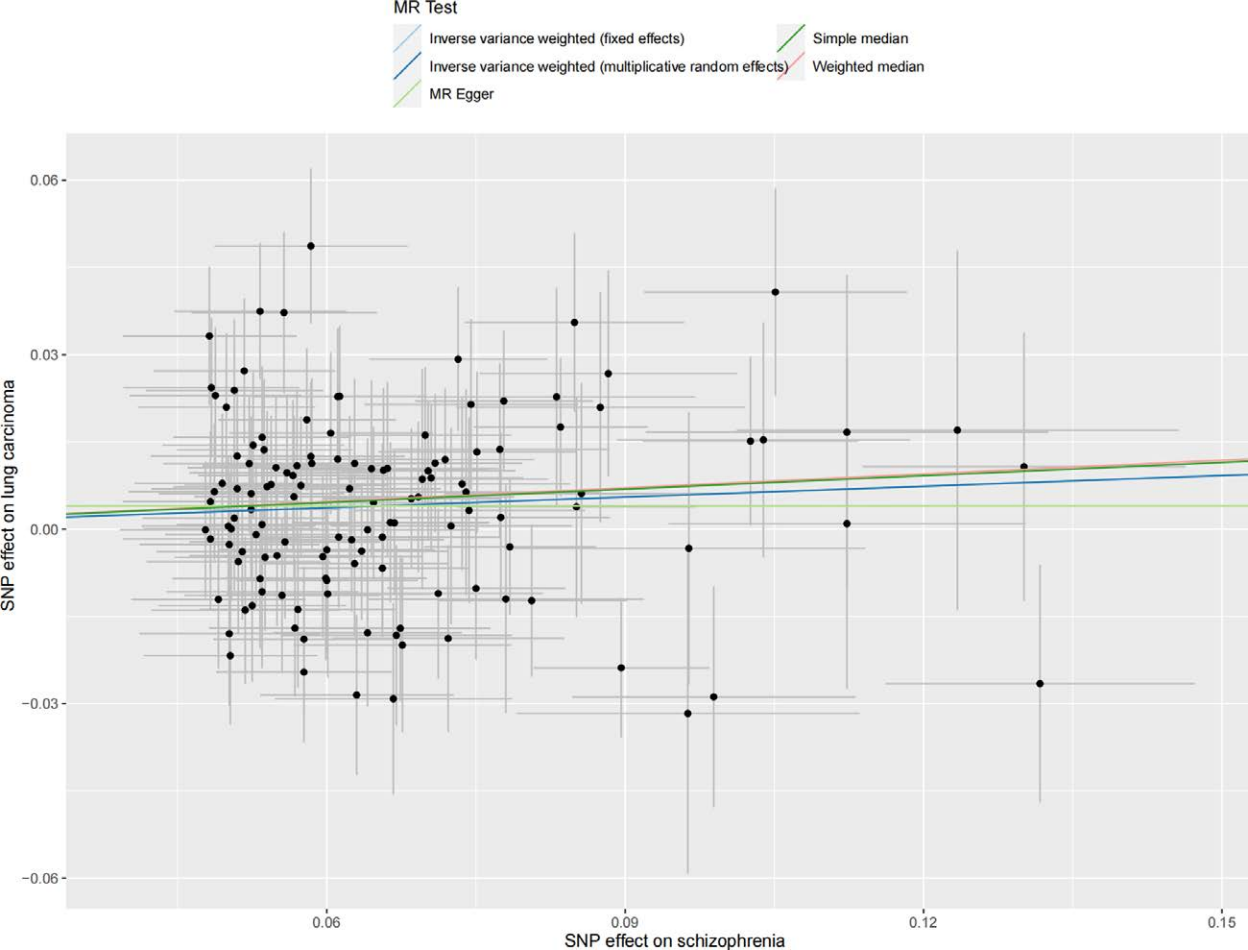
Scatter plot of the MR estimates for the association of schizophrenia with risk of lung carcinoma. MR-PRESSO = MR Pleiotropy Residual Sum and Outlier, SNP = single nucleotide polymorphism.

We extracted 129 SNPs from the outcome GWAS (Table S3, Supplemental Digital Content, http://links.lww.com/MD/L874), and the F-statistics for schizophrenia-related SNPs ranged from 45.04 to 116.28, indicating a strong IVs-exposure association. Our analysis revealed a significant association between genetically schizophrenia and LC. The IVW method demonstrated that schizophrenia was a risk factor for LC, with an odds ratio (OR) of 1.06 [95% confidence interval (CI) 1.02–1.11], and a *P* value of 3.48E-03 (Fig. 2). Consistent results were observed with the Weighted Median method (OR = 1.08, 95% CI 1.02–1.14, *P* = 4.29E-03) and MR-PRESSO methods (OR = 1.06, 95% CI 1.02–1.11, *P* = 4.12E-03), further confirming the robustness of our findings. The corresponding scatter plots are shown in Figure 3.

### 3.2. MDD–LC risk association

Forty SNPs were extracted for further MR analysis, with F-statistics ranging from 83.33 to 232.56. However, the major IVW method did not reveal a significant causal association between MDD and LC (OR = 1.14, 95% CI 0.94–1.39, *P* = .20) (Table S4, Supplemental Digital Content, http://links.lww.com/MD/L875). Similarly, the weighted median and MR-methods also showed non-significant results (*P* = .09, 0.97, respectively), although the direction of the effect for MDD aligned with the main analysis.

After excluding one outlier SNP identified by MRPRESSO (rs198457), our reanalysis included 39 SNPs (Table S3, Supplemental Digital Content, http://links.lww.com/MD/L874). Nevertheless, the major IVW method still did not provide strong evidence for a causal relationship between MDD and LC (OR = 1.18, 95% CI 0.98–1.42, *P* = .07) (Fig. 2). Neither the Weighted Median nor MR-Egger methods yielded statistically significant differences (*P* = .11, 0.86, respectively), and MR-PRESSO showed a marginal result (OR = 1.19, 95% CI 1.00–1.42, *P* = .053). The scatter plot is shown in Figure S1, Supplemental Digital Content, http://links.lww.com/MD/L876.

### 3.3. BD–LC risk association

We utilized 16 SNPs related to BD for additional MR analysis (Table S3, Supplemental Digital Content, http://links.lww.com/MD/L874). The F-statistics for these SNPs ranged from 45.66 to 74.62, indicating a minimal influence from weak instrumental bias. However, both the primary IVW method and complementary approaches provided limited evidence for a causal link between BD and lung carcinoma (OR = 1.07, 95% CI 0.99–1.15, *P* = .09) (Fig. 2). A scatter plot of the MR results is shown in Figure S2, Supplemental Digital Content, http://links.lww.com/MD/L877.

### 3.4. The two-step MR

In the first step, we found significant associations between schizophrenia and certain factors: smoking initiation (beta1 = 0.030, *P* = .005), alcohol consumption (beta1 = 0.010, *P* = .019), and physical activity (beta1 = 0.014, *P* = .003) (Table 2). However, we observed a negative association between BMI and schizophrenia (beta1 = −0.021, *P* = .012), which was the opposite of that of beta0. Consequently, we excluded BMI from the calculation of the mediating proportion.

**Table 2:** The MR effect of schizophrenia on mediators.

Exposure	Outcome	SNPs, n	Methods	Beta	SE	*P* value	Heterogeneity test		Pleiotropy test		
							Q statistic	Q *P* value	Egger intercept	SE	*P* value
Schizophrenia	Smoking Initiation	127	Inverse variance weighted	0.030	0.011	*.005*	461.572	8.45E-40	2.25E-04	0.003	.944
		127	Weighted median	0.016	0.010	*.010*					
		127	MR-Egger	0.026	0.050	.598	461.554	4.41E-40			
Schizophrenia	Alcohol Consumption	126	Inverse variance weighted	0.010	0.004	*.019*	263.720	6.13E-12	1.40E-04	0.001	.916
		126	Weighted median	0.007	0.005	.138					
		126	MR-Egger	0.008	0.021	.697	263.696	4.20E-12			
Schizophrenia	Physical Activity	135	Inverse variance weighted	0.014	0.005	*.003*	275.342	8.24E-12	0.002	0.001	.165
		135	Weighted median	0.010	0.005	.070					
		135	MR-Egger	−0.017	0.023	.464	271.374	1.62E-11			
Schizophrenia	Body Mass Index	135	Inverse variance weighted	−0.021	0.008	*.012*	1006.058	1.47E-133	0.002	0.002	.418
		135	Weighted median	−0.019	0.006	*.002*					
		135	MR-Egger	−0.051	0.039	.188	1001.082	4.64E-133			
Schizophrenia	Type 2 diabetes	123	Inverse variance weighted	1.52E-04	2.89E-04	.598	146.924	.062	−3.86E-05	1.02E-04	.705
		123	Weighted median	2.88E-04	3.98E-04	.469					
		123	MR-Egger	7.63E-04	1.64E-03	.642	146.750	.056			

Italic for *P* < .05 in MR analysis

MR = Mendelian randomization, SE = standard deviation.

In the second step, we confirmed the causal association between these mediators and lung carcinoma (Table 3). Smoking (IVW: beta2 = 0.504, *P* = 1.23E-12) and alcohol consumption (IVW: beta2 = 0.465, *P* = 3.83E-05) showed significant causal effects. MR-Egger and weighted median estimations were aligned with the IVW MR analysis in terms of direction. All three coefficients, beta0, beta1, and beta2, were statistically significant, indicating a causal relationship between exposure and outcome, with the mediator variables partially mediating this relationship. The proportion of each mediator was calculated using the prescribed formula. The results revealed that Smoking and alcohol consumption mediated the effect of schizophrenia on lung carcinoma, accounting for 24.66% and 7.59%, respectively.

**Table 3:** The MR effect of mediators on Lung carcinoma and mediate proportion.

Mediator	Outcome	SNPs, n	Beta2	*P* value for beta2	Beta1	*P* value for beta1	Mediate proportion (%)
Smoking initiation	Lung Carcinoma	79	0.504	*1.23E-12*	0.030	*.005*	24.66
Alcohol consumption	Lung Carcinoma	32	0.465	*3.83E-05*	0.010	*.019*	7.59
Physical activity	Lung Carcinoma	18	−0.730	0.071	0.014	*.003*	–
Body mass index	Lung Carcinoma	410	0.246	*3.09E-07*	-0.021	*.012*	–
Type 2 diabetes	Lung Carcinoma	12	0.134	.974	1.52E-04	.598	**–**

Italic for *P* < .05.

Beta1 exposure’s casual effect on the mediator.

Beta2 mediator’s causal effect on the outcome.

### 3.5. Sensitivity analyses

**Figure 4.**
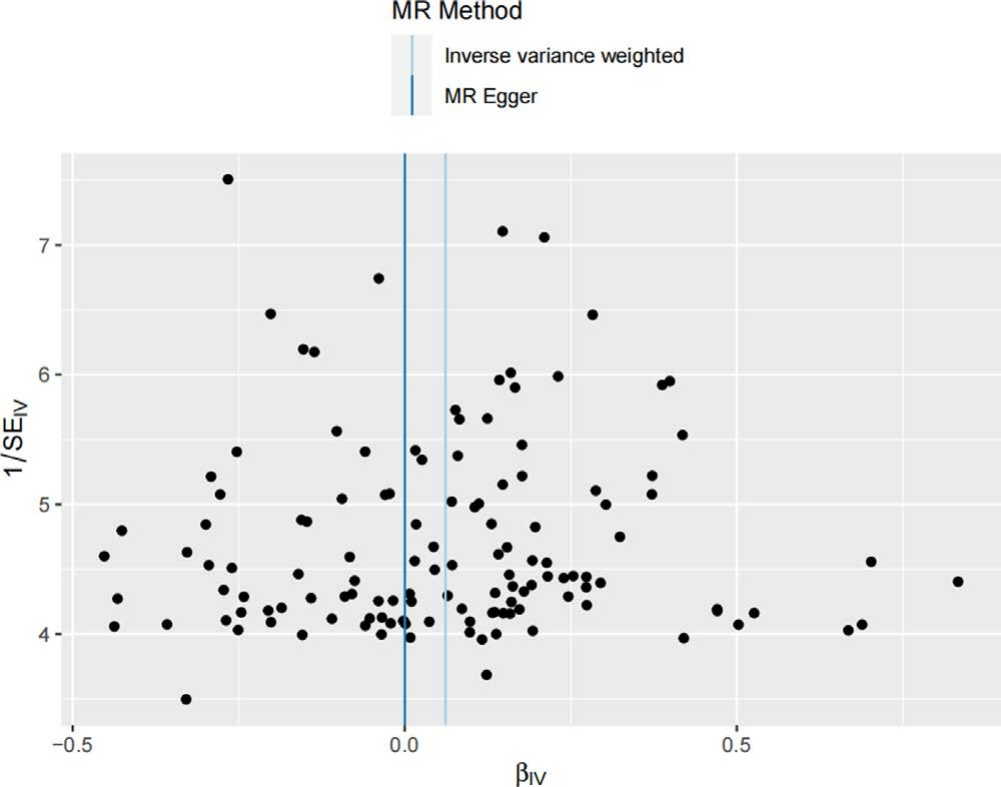
Funnel plot for the causal effect of schizophrenia on lung carcinoma risk. Individual SNP was delineated in the background. SNP = single nucleotide polymorphism.

We used Cochran’s Q test as a heterogeneity test, which demonstrated that several significant differences were noted in the estimated causal associations of exposures with LC risks (Table 4). Consequently, we applied a random model within the IVW method when the *P* value was < .05. The Leave-one-out sensitivity test (Figures S3–5, Supplemental Digital Content, http://links.lww.com/MD/L884; http://links.lww.com/MD/L878; http://links.lww.com/MD/L879) demonstrated minimal alterations when eliminating any schizophrenia, BD, or MDD-related SNP, underscoring the robustness of the MR results. Funnel plots for schizophrenia, MDD, and BD are presented in Figure 4 and Figures S6 and S7, Supplemental Digital Content, http://links.lww.com/MD/L880; http://links.lww.com/MD/L881. In terms of pleiotropy testing, no potential pleiotropy was detected, as evidenced by the MR-Egger intercept *P* values > .05, in all exposure MR analyses (Table 4). Three forest plots for schizophrenia, MDD, and BD are presented in Figures S8–10, Supplemental Digital Content, http://links.lww.com/MD/L885; http://links.lww.com/MD/L882; http://links.lww.com/MD/L883. Additionally, we found no pleiotropic SNP corrected with the MR-PRESSO outlier test among schizophrenia and BD-related SNPs, while one SNPs (rs198457) was detected as an outlier.

**Table 4:** The MR heterogeneity and pleiotropy test.

Exposure		SNPs, n	Heterogeneity		Pleiotropy		
			MR-Egger Q *P* value	Inverse variance weighted Q *P* value	Egger intercept	SE	*P* value
Schizophrenia		129	.012	.014	3.99E-03	6.39E-03	.533
Major depression	Initial MR	40	8.32E-05	1.22E-04	3.29E-03	.019	.8613
	Exclude outlier	39	2.30E-03	3.17E-03	1.91E-03	.017	.912
Bipolar disorder		16	.355	.250	−0.036	.022	.125

MR = Mendelian randomization, SE = standard deviation, SNP = single nucleotide polymorphisms.

### 3.6. Power analysis

We calculated the statistical power of MR using an online statistical tool (https://shiny.cnsgenomics.com/mRnd/).^[42]^ With a type I error of 0.05 and a K value (case composition ratio) of 34.14% for LC, the R^2^_xz_ for schizophrenia was 10.55%. The sample size of the pooled LC data was 85,716, including 29,266 cases and 56,450 controls. Our study had adequate statistical power (80%) to detect an expected odds ratio (OR) of 1.06 for schizophrenia.

## 4. Discussion

In this study, we conducted a two-sample Mendelian randomization analysis to identify a causal relationship between schizophrenia and lung cancer. We adhered to the three major hypotheses of Mendelian randomization and performed a sensitivity analysis to ensure the absence of pleiotropy. We responded to heterogeneity in the analysis by applying the stochastic IVW model. We also performed a two-step Mendelian randomization analysis to detect potential mediators, and found that smoking and alcohol consumption partially mediated the influence of schizophrenia on lung cancer.

Previous studies have reported an increased risk of lung carcinoma among adults with schizophrenia.^[7,8,44]^ A recent study identified a higher risk of lung cancer among women with schizophrenia.^[45]^ We identified schizophrenia as a risk factor for LC (OR = 1.06, 95% CI 1.02–1.11, *P* = 3.48E-03), which corresponds with previous research. However, a meta-analysis involving 12 studies and 496,265 patients found no significant increase in the incidence rate ratio when comparing the incidence of lung cancer between individuals with schizophrenia (SCZ) and the general population.^[46]^ It is hypothesized that this lack of significance may be partially attributed to the protective effect of antipsychotic medication,^[47,48]^ which counteracts the cancer-promoting effect of schizophrenia. Moreover, Individuals with schizophrenia often experience healthcare disparities owing to stigma, physician bias, and poor integration of mental health and medical care. This can result in less comprehensive care, lower-quality diagnoses, and a pseudo-decrease in lung cancer incidence.^[49–51]^ Many lung cancer cases in patients with schizophrenia are likely to be undiagnosed until the late stages or postmortem, leading to latent cases. Additionally, the diagnosis is further complicated by diagnostic overshadowing, where physical symptoms are mistaken for mental illness manifestations.^[49]^ Such misclassification tends to bias hazard estimates downward,^[51]^ and this bias cannot be easily mitigated in observational studies.

In that meta-analysis, the authors acknowledged substantial heterogeneity within the study, which may contribute to the uncertain link between schizophrenia and lung cancer risk. The author also suggested that exploring potential associations between schizophrenia and cancer risk at the biological or genetic level could provide more clarity. Our present results indicate that previous inconsistent findings may partly result from the relatively modest impact of schizophrenia on lung cancer susceptibility (OR 1.06). The prevalence of smoking among individuals with schizophrenia,^[52]^ along with the effects of other confounding variables,^[11]^ complicates the relationship between schizophrenia and lung cancer, making it challenging to identify schizophrenia as a definitive risk factor for lung carcinoma in observational studies.

Accumulating evidence indicates genetic connections between the mechanisms underlying schizophrenia and lung cancer. A recent study on the prognostic and immunomodulatory roles of schizophrenia-associated genes in pan-cancer revealed that PRODH is poorly expressed in lung adenocarcinoma and lung squamous cell cancer, leading to poor prognosis.^[53]^ Additionally, another study identified three independent loci jointly associated with schizophrenia and lung cancer, suggesting a potential downstream polyphenic effect.^[54]^ Our study complements previous research and contributes to a broader understanding of the relationship between schizophrenia and lung cancer.

To explore the mediating factors and mechanisms, we conducted a two-step MR analysis of the various confounding factors. Our findings indicate that smoking can explain 24.66% of the causal relationship between schizophrenia and lung cancer risk, whereas alcohol consumption accounted for 7.59% of the effect. Considering that Individuals with schizophrenia have a notably higher prevalence of cigarette smoking than the general population,^[55]^ which may be linked to the loss of nicotinic receptor expression,^[54,56]^ our findings complement the understanding of the role of smoking in schizophrenia and reinforce the importance of smoking cessation. However, given the common occurrence of alcohol consumption during the lifetime of individuals with schizophrenia,^[57]^ our results encourage patients to abstain from alcohol. Individuals diagnosed with schizophrenia face a significantly elevated risk of lung cancer mortality,^[58]^ yet they undergo fewer lung cancer screenings than the general population.^[6]^ Consequently, our findings emphasize the importance of implementing early screening and intervention strategies for individuals with schizophrenia, particularly for those who are smokers or alcohol consumers.

Major depressive disorder (MDD) is a common condition that affects women at twice the rate of men, with an estimated 1-year prevalence of approximately 6%.^[59]^ Previous studies have suggested a connection between depression and lung cancer incidence.^[60,61]^ However, a recent study found no significant difference in cancer incidence between individuals with MDD and the general population, except for a higher incidence of bronchial and lung cancers in men.^[62]^ An earlier study reported a higher likelihood of depression among patients with lung cancer than among those at initial cancer diagnosis.^[63]^ However, subsequent investigations did not find an elevated incidence of lung cancer in individuals with depression. After excluding the outlier SNP using the MR-PRESSO method, we also found no causal link between MDD and lung cancer (*P* = .07), which aligns with previous research.^[64]^ Nevertheless, it is important to note that MDD is more prevalent in lung cancer patients, significantly affecting their quality of life.^[65]^ Therefore, there should be increased attention and support for patients with lung cancer who experience depression.

Bipolar disorder comprises a spectrum of psychiatric disorders marked by recurring, chronic, and severe episodes of mania or hypomania alongside depressive episodes. A previous study revealed that individuals with schizophrenia or bipolar disorder in the cohort had a 2.6-fold higher overall cancer incidence. The elevated risk was the greatest for lung cancer.^[7]^ However, recent retrospective studies examining its link to carcinoma incidence did not find an elevated occurrence of lung cancer among individuals with BD.^[66,67]^ Consistent with prior research, our MR investigation also suggested that BD did not exert a causal influence on lung carcinoma (*P* = .09).

The strength of our study is its ability to identify a causal relationship between schizophrenia and LC incidence through the application of the MR method. Furthermore, our findings revealed a mediating role of smoking and alcohol consumption in this association. These outcomes underscore the significance of improving LC screening among individuals with mental health conditions and addressing unhealthy behaviors, particularly smoking and alcohol consumption.

Nonetheless, it is essential to acknowledge and address certain limitations in our study. First, the summary GWAS data were derived from individuals of European descent. Therefore, our findings may not be a comprehensive representation of the entire population. Second, we did not perform further subgroup analysis for lung carcinoma, mainly due to the limited number of cases and SNP available within subgroups. This approach aligns with previous clinical research^[7,45,46]^ and the robustness and significance of our results remain intact. Finally, there are potential unmeasured confounding factors, such as the duration and intensity of schizophrenia exposure and the duration of antipsychotic medication intake, which could have influenced our results.

Comprehensive clinical studies with larger sample sizes are warranted to thoroughly investigate the association between schizophrenia and lung cancer risk. Further research is needed to elucidate the mechanisms underlying this relationship. In clinical practice, it is imperative to integrate lung cancer risk assessments into healthcare management plans for individuals with schizophrenia. This should encompass regular lung cancer screening, especially for those who smoke and consume alcohol. Simultaneously, there is a pressing need for health education programs targeting individuals with severe mental illnesses, emphasizing smoking cessation and moderating alcohol intake. Additionally, efforts are needed to remove barriers and improve access to cancer screening in adults with serious mental illnesses. Collaborative initiatives involving psychiatrists, oncologists, and public health experts are essential for the better management of these conditions across different medical domains.

## 5. Conclusions

In conclusion, our two-sample Mendelian randomization analysis revealed that schizophrenia was a risk factor for lung carcinoma. Moreover, we identified smoking and alcohol consumption as the mediating factors in this causal relationship. These findings underscore the importance of improving health management strategies for individuals with schizophrenia, particularly for those who smoke and consume alcohol regularly.

## Author contributions

**Conceptualization:** Xiaohan Chen, Weiyu Shen.

**Data curation:** Shudan Wang.

**Formal analysis:** Xiaohan Chen.

**Funding acquisition:** Weiyu Shen.

**Investigation:** Xiaohan Chen.

**Methodology:** Xiaohan Chen.

**Project administration:** Weiyu Shen.

**Resources:** Weiyu Shen.

**Software:** Shudan Wang.

**Supervision:** Weiyu Shen.

**Validation:** Shudan Wang.

**Visualization:** Xiaohan Chen.

**Writing – original draft:** Xiaohan Chen.

**Writing – review & editing:** Xiaohan Chen, Shudan Wang, Weiyu Shen.

## Supplementary Material









**Figure:**
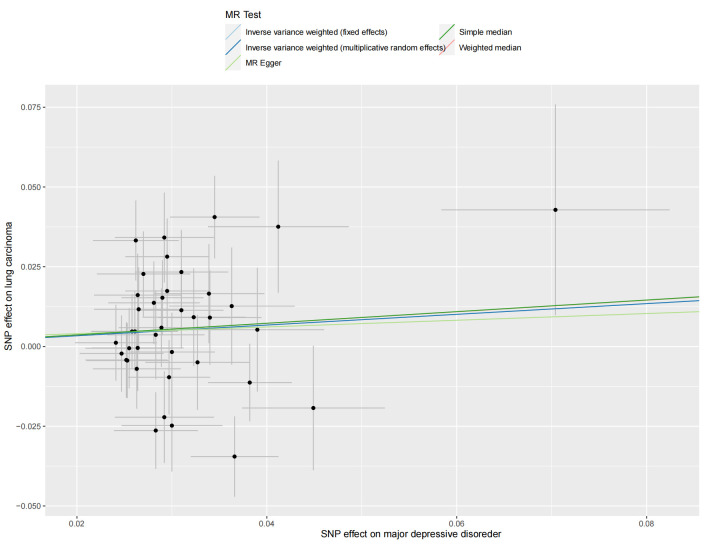


**Figure:**
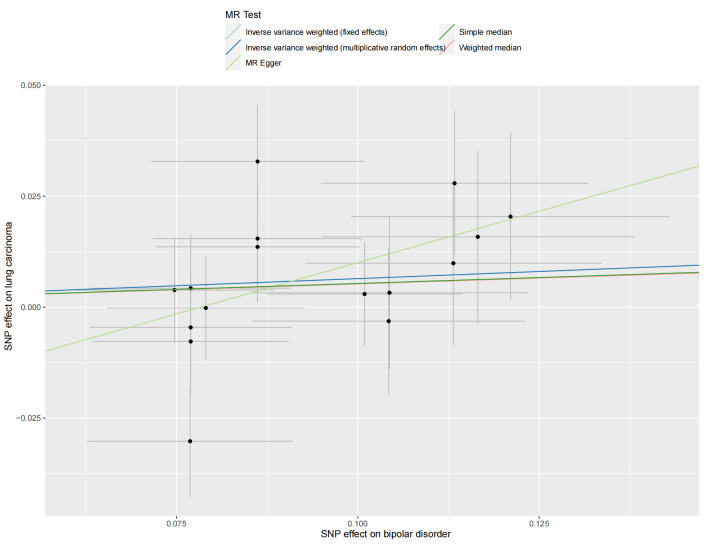


**Figure:**
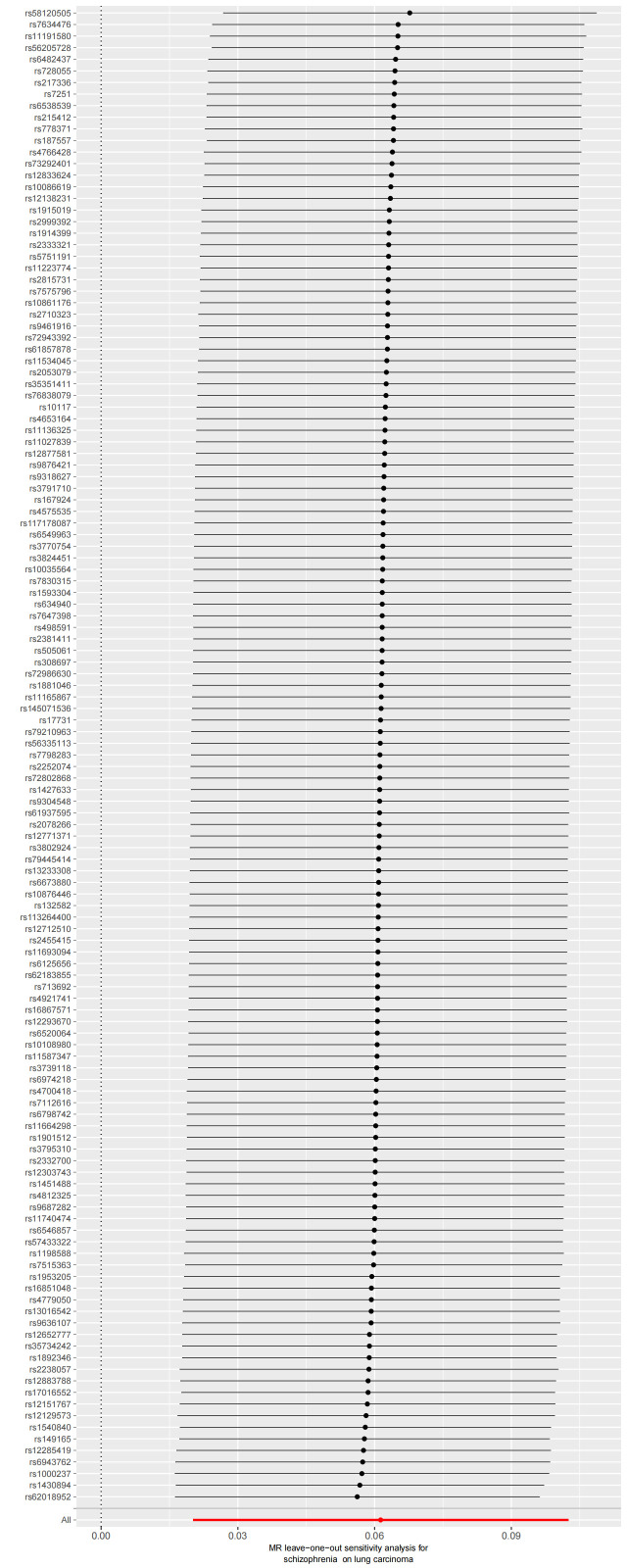


**Figure:**
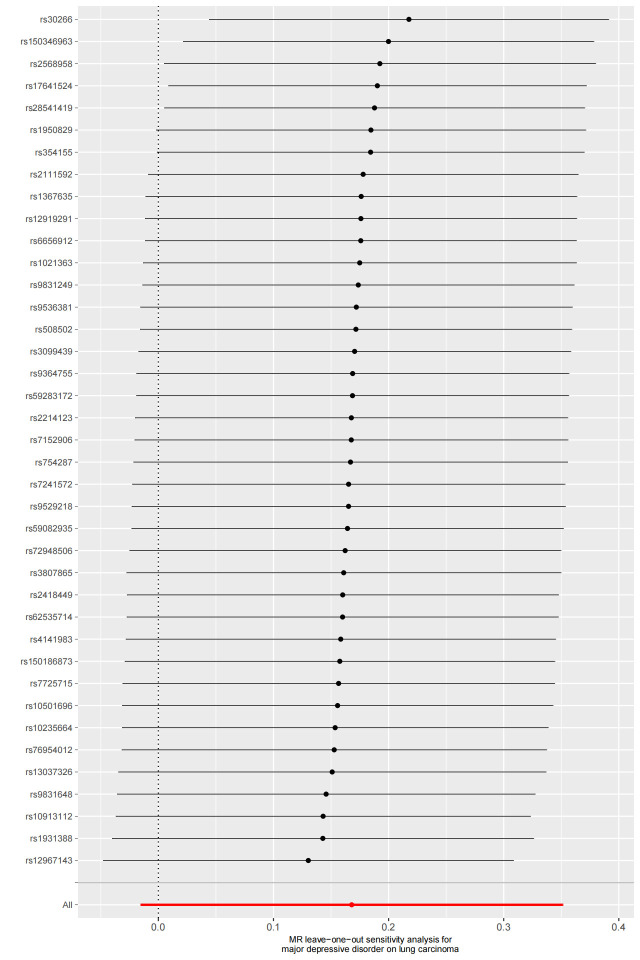


**Figure:**
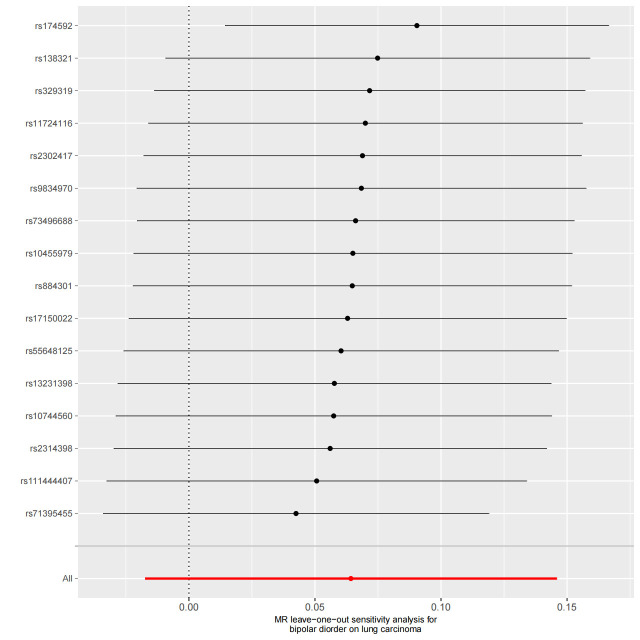


**Figure:**
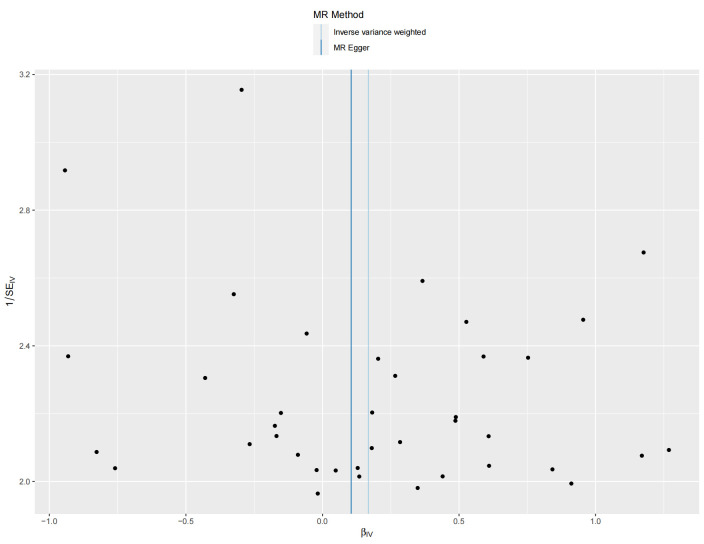


**Figure:**
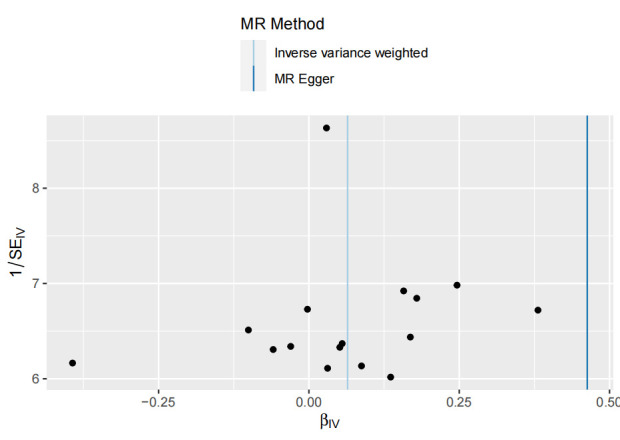


**Figure:**
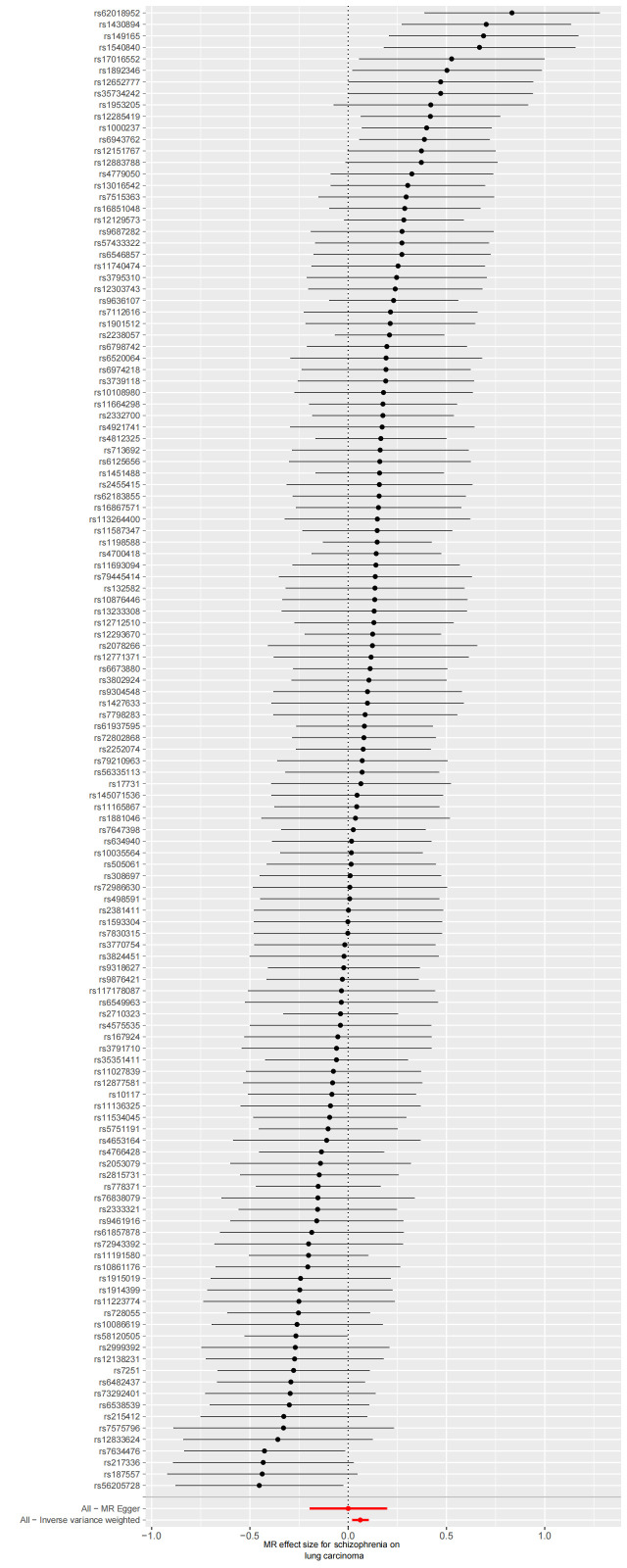


**Figure:**
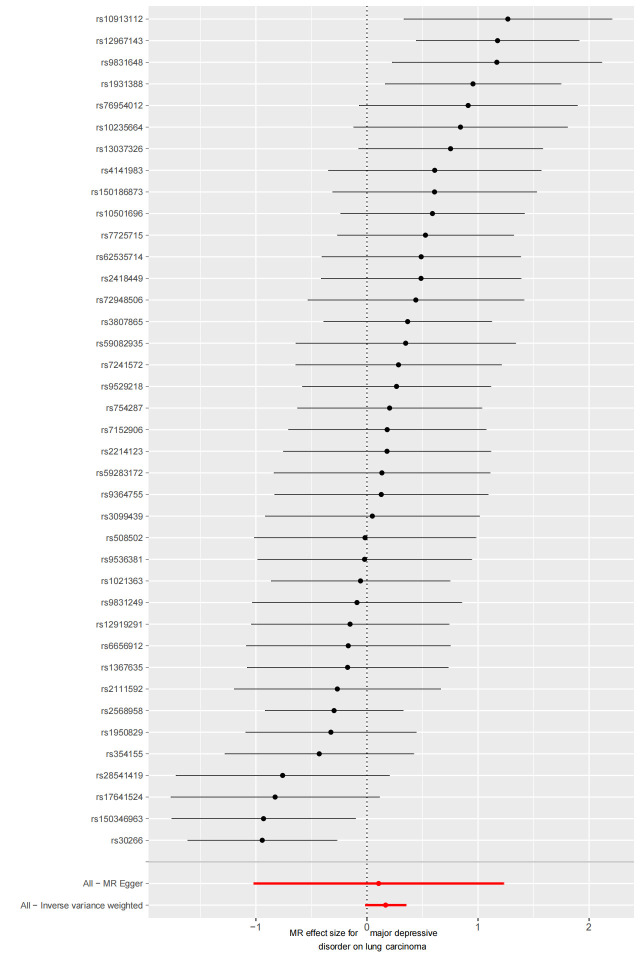


**Figure:**
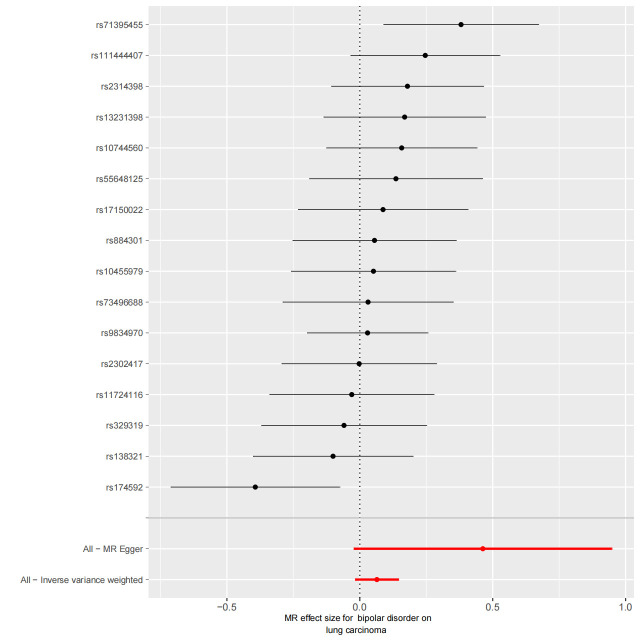

