# Supplementary material for: The causal relationship between severe mental illness and risk of lung carcinoma
Source: Medicine (Baltimore). 2024 Mar 15;103(11):e37355. doi: 10.1097/MD.0000000000037355 (PMC10939700; doi:10.1097/MD.0000000000037355)
Supplement: Supplementary file 1 [file medi-103-e37355-s001.docx]

| **Table S1 Details of studies included in confounders and mediators of SMI** | | | | | |
| --- | --- | --- | --- | --- | --- |
|  |  |  |  |  |  |
| Trait | GWAS ID | Consortium | Year | Sample size | Number of SNPs |
| smoking initiation | ieu-b-4877 | GSCAN | 2019 | 607,291 | 11,802,365 |
| Alcoholic drinks per week | ieu-b-73 | GWAS and Sequencing Consortium of Alcohol and Nicotine use | 2022 | 83,626 | 7,914,362 |
| Physical activity | ebi-a-GCST006097 | NA | 2018 | 377,234 | 11,808,007 |
| Body mass index (BMI) | ukb-b-19953 | MRC-IEU | 2018 | 461,460 | 9,851,867 |
| type 2 diabetes | ukb-b-13806 | MRC-IEU | 2018 | 462,933 | 9,851,867 |
| SMI severe mental illness, NA not available,SNP single nucleotide polymorphism. |  |  |  |  |  |
