# Supplementary material for: The causal relationship between severe mental illness and risk of lung carcinoma
Source: Medicine (Baltimore). 2024 Mar 15;103(11):e37355. doi: 10.1097/MD.0000000000037355 (PMC10939700; doi:10.1097/MD.0000000000037355)
Supplement: Supplementary file 2 [file medi-103-e37355-s002.docx]

| **Table S2 Genome-wide significant SNPs loci association with schizophrenia, MDD, and BD for Mendelian randomization on lung carcinoma risk** | | | | | | | | | | | |
| --- | --- | --- | --- | --- | --- | --- | --- | --- | --- | --- | --- |
|  |  |  |  |  |  |  |  |  |  |  |  |
| **SNP** | **chr** | **pos** | **effect_allele** | **other_allele** | **BETA** | **SE** | **P-value** | **EAF** | **samplesize** | **R2** | **F-statistic** |
| **Schizophrenia** | | | | | | | | | | | |
| rs6715366 | 2 | 2327295 | G | A | -0.054097222 | 0.0097 | 2.49E-08 | 7.23E-01 | 130644 | 0.00078849 | 103.0912053 |
| rs2532240 | 17 | 44265839 | C | T | 0.060803429 | 0.0091 | 2.58E-11 | 6.10E-01 | 130644 | 0.000840435 | 109.8884276 |
| rs1892346 | 1 | 66331478 | T | A | -0.048402739 | 0.0088 | 3.56E-08 | 4.28E-01 | 130644 | 0.000869061 | 113.634624 |
| rs7515363 | 1 | 200414959 | C | T | 0.053502852 | 0.0089 | 1.84E-09 | 3.89E-01 | 130644 | 0.000859305 | 112.3578305 |
| rs3739118 | 2 | 201253769 | G | A | 0.057003957 | 0.0095 | 2.36E-09 | 7.19E-01 | 130644 | 0.000805076 | 105.2615464 |
| rs4653164 | 1 | 36627542 | C | T | -0.051103839 | 0.0092 | 3.08E-08 | 3.23E-01 | 130644 | 0.000831307 | 108.6939882 |
| rs56335113 | 1 | 30427639 | A | G | 0.064701008 | 0.0094 | 6.02E-12 | 3.16E-01 | 130644 | 0.000813634 | 106.3813501 |
| rs1915019 | 8 | 89283689 | A | G | 0.057098412 | 0.0098 | 6.57E-09 | 2.65E-01 | 130644 | 0.000780451 | 102.0392542 |
| rs308697 | 3 | 161487491 | C | A | 0.050103587 | 0.0087 | 8.83E-09 | 5.74E-01 | 130644 | 0.000879041 | 114.9407691 |
| rs12833624 | 12 | 124476873 | C | T | -0.050199156 | 0.009 | 2.77E-08 | 6.46E-01 | 130644 | 0.000849765 | 111.1094101 |
| rs132582 | 22 | 39988175 | C | T | 0.050997251 | 0.0086 | 3.26E-09 | 4.71E-01 | 130644 | 0.000889254 | 116.2772897 |
| rs167924 | 3 | 107379837 | A | G | -0.050199156 | 0.009 | 2.34E-08 | 3.59E-01 | 130644 | 0.000849765 | 111.1094101 |
| rs72943392 | 11 | 81178838 | G | C | -0.053495718 | 0.0096 | 2.39E-08 | 7.07E-01 | 130644 | 0.000796697 | 104.165072 |
| rs9876421 | 3 | 36848316 | C | T | -0.06250326 | 0.0092 | 9.19E-12 | 6.45E-01 | 130644 | 0.000831307 | 108.6939882 |
| rs6549963 | 3 | 30044778 | T | C | 0.048304332 | 0.0088 | 4.31E-08 | 5.96E-01 | 130644 | 0.000869061 | 113.634624 |
| rs6538539 | 12 | 95195293 | G | T | 0.056796126 | 0.0086 | 4.43E-11 | 4.62E-01 | 130644 | 0.000889254 | 116.2772897 |
| rs7575796 | 2 | 97746526 | A | G | 0.096300598 | 0.0172 | 2.07E-08 | 9.17E-01 | 130644 | 0.000444825 | 58.13864484 |
| rs10086619 | 8 | 111580570 | A | G | -0.072205168 | 0.0116 | 4.97E-10 | 8.31E-01 | 130644 | 0.000659426 | 86.20557683 |
| rs4702 | 15 | 91426560 | G | A | 0.084304383 | 0.0089 | 2.79E-21 | 4.61E-01 | 130644 | 0.000859305 | 112.3578305 |
| rs11136325 | 8 | 144870701 | G | A | 0.053796658 | 0.0091 | 3.05E-09 | 4.36E-01 | 130644 | 0.000840435 | 109.8884276 |
| rs12303743 | 12 | 72259954 | G | C | -0.087498768 | 0.0145 | 1.59E-09 | 8.98E-01 | 130644 | 0.00052761 | 68.96446146 |
| rs217336 | 6 | 84341803 | C | A | 0.050303305 | 0.0087 | 8.05E-09 | 5.83E-01 | 130644 | 0.000879041 | 114.9407691 |
| rs3824451 | 9 | 101071522 | T | C | -0.065595079 | 0.0118 | 2.54E-08 | 8.38E-01 | 130644 | 0.000648257 | 84.74446536 |
| rs2381411 | 9 | 36319928 | T | C | -0.050398958 | 0.0088 | 1.25E-08 | 5.86E-01 | 130644 | 0.000869061 | 113.634624 |
| rs12293670 | 11 | 124612932 | A | G | 0.070495742 | 0.0092 | 1.56E-14 | 6.78E-01 | 130644 | 0.000831307 | 108.6939882 |
| rs11740474 | 5 | 153680747 | A | T | -0.053696179 | 0.0088 | 1.13E-09 | 5.75E-01 | 130644 | 0.000869061 | 113.634624 |
| rs72802868 | 5 | 152235215 | G | T | 0.069199517 | 0.0096 | 4.55E-13 | 7.24E-01 | 130644 | 0.000796697 | 104.165072 |
| rs12652777 | 5 | 155775075 | T | C | 0.048799688 | 0.0086 | 1.52E-08 | 4.89E-01 | 130644 | 0.000889254 | 116.2772897 |
| rs149165 | 16 | 58659307 | T | G | 0.048199514 | 0.0087 | 3.01E-08 | 5.69E-01 | 130644 | 0.000879041 | 114.9407691 |
| rs12771371 | 10 | 54063083 | G | A | 0.052402679 | 0.0093 | 1.94E-08 | 6.97E-01 | 130644 | 0.000822376 | 107.5252356 |
| rs7830315 | 8 | 65608361 | T | C | -0.04780465 | 0.0086 | 3.08E-08 | 4.78E-01 | 130644 | 0.000889254 | 116.2772897 |
| rs6984242 | 8 | 60700469 | G | A | 0.054696497 | 0.0087 | 3.86E-10 | 4.13E-01 | 130644 | 0.000879041 | 114.9407691 |
| rs58120505 | 7 | 2029867 | T | C | 0.089603016 | 0.0088 | 2.24E-24 | 6.02E-01 | 130644 | 0.000869061 | 113.634624 |
| rs17731 | 10 | 3821561 | G | A | -0.052399169 | 0.0089 | 4.37E-09 | 6.20E-01 | 130644 | 0.000859305 | 112.3578305 |
| rs4766428 | 12 | 110723245 | C | T | -0.075003758 | 0.0089 | 3.93E-17 | 5.39E-01 | 130644 | 0.000859305 | 112.3578305 |
| rs7251 | 19 | 50162909 | C | G | 0.064100925 | 0.0094 | 8.29E-12 | 6.82E-01 | 130644 | 0.000813634 | 106.3813501 |
| rs2999392 | 14 | 51655145 | C | T | -0.051798685 | 0.0094 | 3.05E-08 | 2.99E-01 | 130644 | 0.000813634 | 106.3813501 |
| rs2333321 | 4 | 176859992 | A | G | 0.071203759 | 0.0105 | 1.25E-11 | 2.18E-01 | 130644 | 0.000728458 | 95.23663726 |
| rs1427633 | 4 | 167974142 | G | C | 0.048304332 | 0.0088 | 4.10E-08 | 4.19E-01 | 130644 | 0.000869061 | 113.634624 |
| rs3791710 | 2 | 212290048 | T | C | 0.060003252 | 0.0108 | 3.02E-08 | 8.04E-01 | 130644 | 0.000708238 | 92.59117511 |
| rs6943762 | 7 | 86403263 | T | C | 0.105098481 | 0.0132 | 1.57E-15 | 8.83E-01 | 130644 | 0.000579542 | 75.756416 |
| rs13233308 | 7 | 87244960 | C | T | 0.048704446 | 0.0086 | 1.75E-08 | 5.26E-01 | 130644 | 0.000889254 | 116.2772897 |
| rs2252074 | 7 | 104594253 | T | G | -0.068503705 | 0.0088 | 6.19E-15 | 5.90E-01 | 130644 | 0.000869061 | 113.634624 |
| rs16851048 | 1 | 177276006 | T | C | -0.074497279 | 0.0107 | 4.15E-12 | 7.92E-01 | 130644 | 0.000714852 | 93.4565132 |
| rs11027839 | 11 | 24389235 | A | C | -0.051503843 | 0.0086 | 2.4E-09 | 4.88E-01 | 130644 | 0.000889254 | 116.2772897 |
| rs778371 | 2 | 233743109 | A | G | -0.080602865 | 0.0095 | 1.5E-17 | 6.99E-01 | 130644 | 0.000805076 | 105.2615464 |
| rs4575535 | 16 | 89559297 | A | G | -0.055798163 | 0.0096 | 5.77E-09 | 2.81E-01 | 130644 | 0.000796697 | 104.165072 |
| rs10117 | 5 | 137892170 | G | A | 0.054999418 | 0.0088 | 4.66E-10 | 6.13E-01 | 130644 | 0.000869061 | 113.634624 |
| rs9687282 | 5 | 139065988 | T | G | -0.05259941 | 0.0091 | 7.33E-09 | 6.50E-01 | 130644 | 0.000840435 | 109.8884276 |
| rs7798283 | 7 | 133128127 | T | G | 0.074002967 | 0.0134 | 3.49E-08 | 8.75E-01 | 130644 | 0.000570897 | 74.62572323 |
| rs728055 | 7 | 137072531 | T | A | 0.067396932 | 0.009 | 8.85E-14 | 6.58E-01 | 130644 | 0.000849765 | 111.1094101 |
| rs1593304 | 7 | 131619847 | A | G | -0.064101283 | 0.0111 | 7.45E-09 | 1.93E-01 | 130644 | 0.000689109 | 90.08871092 |
| rs9461916 | 6 | 33796794 | T | C | -0.053295297 | 0.0088 | 1.64E-09 | 3.88E-01 | 130644 | 0.000869061 | 113.634624 |
| rs11693094 | 2 | 185601420 | C | T | 0.054402954 | 0.0087 | 4.29E-10 | 5.56E-01 | 130644 | 0.000879041 | 114.9407691 |
| rs12129573 | 1 | 73768366 | C | A | -0.077799392 | 0.0089 | 2.28E-18 | 6.16E-01 | 130644 | 0.000859305 | 112.3578305 |
| rs215412 | 4 | 23423586 | G | A | -0.057703268 | 0.0091 | 2.69E-10 | 6.61E-01 | 130644 | 0.000840435 | 109.8884276 |
| rs7647398 | 3 | 180733150 | C | T | 0.077497934 | 0.0109 | 1.07E-12 | 8.11E-01 | 130644 | 0.000701745 | 91.74171479 |
| rs1430894 | 3 | 17868759 | C | T | -0.053295297 | 0.0086 | 6.15E-10 | 5.06E-01 | 130644 | 0.000889254 | 116.2772897 |
| rs5751191 | 22 | 42370991 | T | C | -0.065595079 | 0.0086 | 3E-14 | 4.84E-01 | 130644 | 0.000889254 | 116.2772897 |
| rs12151767 | 2 | 198274929 | G | A | 0.061104507 | 0.0086 | 1.31E-12 | 5.24E-01 | 130644 | 0.000889254 | 116.2772897 |
| rs1451488 | 2 | 199990107 | A | G | -0.070894679 | 0.0087 | 4.47E-16 | 4.32E-01 | 130644 | 0.000879041 | 114.9407691 |
| rs3770754 | 2 | 37575381 | C | G | 0.052896009 | 0.0091 | 5.35E-09 | 6.45E-01 | 130644 | 0.000840435 | 109.8884276 |
| rs4812325 | 20 | 37485458 | G | A | -0.071904248 | 0.0089 | 8.96E-16 | 3.70E-01 | 130644 | 0.000859305 | 112.3578305 |
| rs13016542 | 2 | 145183851 | T | C | 0.088303875 | 0.0129 | 8.28E-12 | 8.76E-01 | 130644 | 0.000593012 | 77.51819312 |
| rs500102 | 9 | 77358745 | T | C | 0.051700212 | 0.0088 | 4.87E-09 | 4.13E-01 | 130644 | 0.000869061 | 113.634624 |
| rs2078266 | 9 | 138378856 | A | G | 0.069600687 | 0.0126 | 2.94E-08 | 1.78E-01 | 130644 | 0.000607122 | 79.36386438 |
| rs11191580 | 10 | 104906211 | T | C | 0.131703473 | 0.0155 | 1.77E-17 | 9.20E-01 | 130644 | 0.000493588 | 64.51514137 |
| rs2815731 | 6 | 73155285 | C | A | 0.060003252 | 0.0091 | 4.39E-11 | 6.60E-01 | 130644 | 0.000840435 | 109.8884276 |
| rs10876446 | 12 | 53760710 | G | C | -0.054002223 | 0.0094 | 1.03E-08 | 6.74E-01 | 130644 | 0.000813634 | 106.3813501 |
| rs61937595 | 12 | 57682956 | C | T | 0.130098005 | 0.0162 | 1.15E-15 | 9.17E-01 | 130644 | 0.00047227 | 61.72745008 |
| rs73292401 | 17 | 12875908 | T | A | -0.06760455 | 0.0109 | 5.48E-10 | 7.99E-01 | 130644 | 0.000701745 | 91.74171479 |
| rs57433322 | 17 | 19141582 | C | G | 0.083099569 | 0.0139 | 1.99E-09 | 8.85E-01 | 130644 | 0.000550372 | 71.94134469 |
| rs187557 | 5 | 106766340 | C | T | 0.066695568 | 0.0119 | 2.03E-08 | 1.63E-01 | 130644 | 0.000642812 | 84.03232699 |
| rs1901512 | 5 | 101723875 | T | C | 0.058400975 | 0.0094 | 5.72E-10 | 3.18E-01 | 130644 | 0.000813634 | 106.3813501 |
| rs10861176 | 12 | 104631552 | G | A | -0.055502139 | 0.0098 | 1.59E-08 | 2.57E-01 | 130644 | 0.000780451 | 102.0392542 |
| rs2455415 | 13 | 38860697 | C | T | -0.049494912 | 0.0088 | 1.69E-08 | 5.77E-01 | 130644 | 0.000869061 | 113.634624 |
| rs10035564 | 5 | 45252500 | A | G | -0.066802415 | 0.0092 | 4.38E-13 | 6.50E-01 | 130644 | 0.000831307 | 108.6939882 |
| rs1540840 | 14 | 99733384 | G | C | 0.055699572 | 0.0093 | 2.21E-09 | 5.42E-01 | 130644 | 0.000822376 | 107.5252356 |
| rs17194490 | 3 | 2547786 | G | T | -0.078199408 | 0.0116 | 1.8E-11 | 8.27E-01 | 130644 | 0.000659426 | 86.20557683 |
| rs61857878 | 10 | 92789488 | A | T | 0.060097424 | 0.0102 | 4.44E-09 | 7.60E-01 | 130644 | 0.000749868 | 98.03771483 |
| rs1881046 | 2 | 156835793 | G | T | 0.05070262 | 0.0092 | 3.39E-08 | 6.70E-01 | 130644 | 0.000831307 | 108.6939882 |
| rs79210963 | 7 | 24717969 | T | C | -0.085601462 | 0.0137 | 4.14E-10 | 8.84E-01 | 130644 | 0.000558403 | 72.9915833 |
| rs12285419 | 11 | 46343189 | C | A | -0.084904507 | 0.011 | 1.05E-14 | 8.00E-01 | 130644 | 0.00069537 | 90.9076992 |
| rs634940 | 6 | 93077500 | G | T | -0.066396246 | 0.0099 | 1.78E-11 | 7.37E-01 | 130644 | 0.000772573 | 101.0085547 |
| rs117178087 | 6 | 96464060 | C | T | 0.096400494 | 0.0177 | 4.89E-08 | 9.39E-01 | 130644 | 0.000432264 | 56.49631024 |
| rs9304548 | 18 | 27500959 | C | A | 0.056701643 | 0.01 | 1.59E-08 | 2.61E-01 | 130644 | 0.000764853 | 99.99846912 |
| rs2710323 | 3 | 52815905 | T | C | 0.078404443 | 0.0086 | 1.23E-19 | 5.31E-01 | 130644 | 0.000889254 | 116.2772897 |
| rs7634476 | 3 | 136398387 | A | G | -0.057703268 | 0.0088 | 5.46E-11 | 3.97E-01 | 130644 | 0.000869061 | 113.634624 |
| rs4779050 | 15 | 83368738 | T | G | 0.057995286 | 0.0089 | 7.27E-11 | 3.81E-01 | 130644 | 0.000859305 | 112.3578305 |
| rs6673880 | 1 | 2373168 | A | G | -0.062301026 | 0.0091 | 7.2E-12 | 4.92E-01 | 130644 | 0.000840435 | 109.8884276 |
| rs3795310 | 1 | 8431607 | C | T | 0.050997251 | 0.0087 | 5.75E-09 | 5.43E-01 | 130644 | 0.000879041 | 114.9407691 |
| rs4921741 | 8 | 17070926 | A | G | -0.055999086 | 0.0098 | 1.21E-08 | 7.29E-01 | 130644 | 0.000780451 | 102.0392542 |
| rs35351411 | 15 | 61872197 | A | C | -0.063504389 | 0.0087 | 2.21E-13 | 4.39E-01 | 130644 | 0.000879041 | 114.9407691 |
| rs2332700 | 14 | 72417326 | C | G | 0.075098196 | 0.0099 | 3.88E-14 | 2.58E-01 | 130644 | 0.000772573 | 101.0085547 |
| rs1000237 | 19 | 19518316 | T | A | -0.073205303 | 0.0089 | 2.8E-16 | 6.27E-01 | 130644 | 0.000859305 | 112.3578305 |
| rs72986630 | 19 | 11849736 | C | T | -0.112295619 | 0.0179 | 3.59E-10 | 9.26E-01 | 130644 | 0.000427437 | 55.86506655 |
| rs12138231 | 1 | 150115398 | T | A | -0.066994869 | 0.0116 | 7.99E-09 | 1.71E-01 | 130644 | 0.000659426 | 86.20557683 |
| rs11587347 | 1 | 239198959 | C | G | -0.103894923 | 0.0147 | 1.53E-12 | 8.95E-01 | 130644 | 0.000520436 | 68.02616947 |
| rs145071536 | 1 | 243793012 | T | C | -0.085100477 | 0.012 | 1.62E-12 | 7.99E-01 | 130644 | 0.000637459 | 83.3320576 |
| rs6482437 | 10 | 18726326 | A | C | -0.098903629 | 0.0142 | 3.33E-12 | 9.94E-02 | 130644 | 0.000538751 | 70.42145713 |
| rs16867571 | 5 | 88743219 | A | G | 0.065703467 | 0.0104 | 2.68E-10 | 7.78E-01 | 130644 | 0.000735458 | 96.15237416 |
| rs11534045 | 7 | 110935979 | G | A | 0.062796379 | 0.0093 | 1.4E-11 | 6.86E-01 | 130644 | 0.000822376 | 107.5252356 |
| rs6974218 | 7 | 110056000 | A | C | 0.054895299 | 0.0089 | 6.8E-10 | 6.34E-01 | 130644 | 0.000859305 | 112.3578305 |
| rs1914399 | 7 | 71770973 | C | G | 0.049104401 | 0.0087 | 1.40E-08 | 4.89E-01 | 130644 | 0.000879041 | 114.9407691 |
| rs4700418 | 5 | 60621839 | C | G | -0.070197168 | 0.0087 | 5.37E-16 | 4.89E-01 | 130644 | 0.000879041 | 114.9407691 |
| rs113264400 | 20 | 62150128 | T | C | -0.112295619 | 0.0202 | 2.87E-08 | 9.48E-01 | 130644 | 0.000378787 | 49.50419263 |
| rs13011472 | 2 | 57961602 | C | G | -0.070401005 | 0.0087 | 4.28E-16 | 4.98E-01 | 130644 | 0.000879041 | 114.9407691 |
| rs9636107 | 18 | 53200117 | A | G | -0.069896851 | 0.0086 | 5.12E-16 | 5.06E-01 | 130644 | 0.000889254 | 116.2772897 |
| rs1953205 | 14 | 30300361 | T | A | -0.049904785 | 0.0089 | 2.22E-08 | 5.06E-01 | 130644 | 0.000859305 | 112.3578305 |
| rs12883788 | 14 | 33303540 | C | T | -0.061301101 | 0.0087 | 1.86E-12 | 5.27E-01 | 130644 | 0.000879041 | 114.9407691 |
| rs2053079 | 19 | 30987423 | A | G | -0.059898635 | 0.0101 | 3.01E-09 | 7.54E-01 | 130644 | 0.000757286 | 99.00838527 |
| rs505061 | 9 | 22767164 | C | A | -0.053495718 | 0.0086 | 5.8E-10 | 4.95E-01 | 130644 | 0.000889254 | 116.2772897 |
| rs12877581 | 13 | 74325499 | G | C | -0.059601395 | 0.0099 | 1.8E-09 | 7.16E-01 | 130644 | 0.000772573 | 101.0085547 |
| rs9318627 | 13 | 79930079 | A | C | 0.061198575 | 0.0088 | 4.35E-12 | 6.13E-01 | 130644 | 0.000869061 | 113.634624 |
| rs6546857 | 2 | 73837955 | A | G | -0.060397773 | 0.0102 | 2.74E-09 | 7.57E-01 | 130644 | 0.000749868 | 98.03771483 |
| rs17016552 | 2 | 79433742 | C | G | 0.051700212 | 0.0091 | 1.20E-08 | 6.61E-01 | 130644 | 0.000840435 | 109.8884276 |
| rs1198588 | 1 | 98552832 | A | T | -0.102597669 | 0.0108 | 1.73E-21 | 1.94E-01 | 130644 | 0.000708238 | 92.59117511 |
| rs11165867 | 1 | 97878068 | C | T | -0.074303376 | 0.0116 | 1.3E-10 | 8.29E-01 | 130644 | 0.000659426 | 86.20557683 |
| rs56205728 | 15 | 40567237 | G | A | -0.0630037 | 0.0097 | 1.01E-10 | 7.00E-01 | 130644 | 0.00078849 | 103.0912053 |
| rs62018952 | 15 | 44080737 | T | C | -0.058402717 | 0.0097 | 1.94E-09 | 2.64E-01 | 130644 | 0.00078849 | 103.0912053 |
| rs62183855 | 2 | 172956449 | A | C | 0.066096682 | 0.0111 | 2.66E-09 | 8.14E-01 | 130644 | 0.000689109 | 90.08871092 |
| rs10108980 | 8 | 4191446 | C | T | -0.062801364 | 0.0106 | 2.73E-09 | 7.85E-01 | 130644 | 0.000721591 | 94.33817842 |
| rs11664298 | 18 | 77578986 | G | A | -0.077399537 | 0.0108 | 8.94E-13 | 7.94E-01 | 130644 | 0.000708238 | 92.59117511 |
| rs76838079 | 18 | 77633271 | C | T | -0.078004786 | 0.0138 | 1.53E-08 | 8.49E-01 | 130644 | 0.000554358 | 72.46265878 |
| rs11223774 | 11 | 134247315 | A | G | 0.052497569 | 0.0094 | 2.74E-08 | 3.01E-01 | 130644 | 0.000813634 | 106.3813501 |
| rs3802924 | 11 | 133827733 | A | C | 0.073603559 | 0.0108 | 9.58E-12 | 8.05E-01 | 130644 | 0.000708238 | 92.59117511 |
| rs7112616 | 11 | 130805334 | T | C | 0.05220338 | 0.0086 | 1.52E-09 | 5.15E-01 | 130644 | 0.000889254 | 116.2772897 |
| rs79445414 | 8 | 33863561 | T | C | -0.123400031 | 0.0222 | 2.80E-08 | 9.56E-01 | 130644 | 0.000344673 | 45.04435546 |
| rs6520064 | 22 | 50294469 | A | G | -0.058498134 | 0.0106 | 3.58E-08 | 7.87E-01 | 130644 | 0.000721591 | 94.33817842 |
| rs713692 | 22 | 51109735 | G | A | -0.056602098 | 0.0095 | 2.67E-09 | 2.99E-01 | 130644 | 0.000805076 | 105.2615464 |
| rs6798742 | 3 | 63903759 | A | G | -0.061099109 | 0.0093 | 4.57E-11 | 6.80E-01 | 130644 | 0.000822376 | 107.5252356 |
| rs498591 | 9 | 14509105 | A | T | -0.072495427 | 0.0121 | 2.11E-09 | 8.47E-01 | 130644 | 0.000632194 | 82.64336291 |
| rs2238057 | 12 | 2384005 | T | G | -0.083501181 | 0.0087 | 8.5E-22 | 5.68E-01 | 130644 | 0.000879041 | 114.9407691 |
| rs12712510 | 2 | 22749726 | T | C | 0.057400607 | 0.0087 | 5.14E-11 | 4.88E-01 | 130644 | 0.000879041 | 114.9407691 |
| rs35734242 | 4 | 706700 | T | C | -0.050703994 | 0.0089 | 1.37E-08 | 5.62E-01 | 130644 | 0.000859305 | 112.3578305 |
| rs6125656 | 20 | 48090779 | G | A | -0.064495855 | 0.0111 | 6.29E-09 | 8.09E-01 | 130644 | 0.000689109 | 90.08871092 |
| **SNP** |  |  | **effect_allele** | **other_allele** | **BETA** | **SE** | **P-value** | **EAF** | **samplesize** | **R2** | **F-statistic** |
| **Major Depression Disorder** | | | | | | | | | | | |
| rs59283172 |  |  | A | G | -0.039 | 0.007 | 2.41E-08 | 1.08E-01 | 807553 | 0.00017687 | 142.8567891 |
| rs7152906 |  |  | T | C | -0.0258 | 0.0043 | 1.87E-09 | 4.80E-01 | 807553 | 0.000287896 | 232.5575636 |
| rs3099439 |  |  | T | C | -0.0241 | 0.0043 | 2.78E-08 | 5.35E-01 | 807553 | 0.000287896 | 232.5575636 |
| rs13037326 |  |  | T | C | 0.031 | 0.0049 | 2.4E-10 | 2.60E-01 | 807553 | 0.000252652 | 204.0811272 |
| rs2111592 |  |  | A | G | 0.0263 | 0.0046 | 1.35E-08 | 3.14E-01 | 807553 | 0.000269125 | 217.390766 |
| rs2876520 |  |  | C | G | -0.026 | 0.0043 | 2.24E-09 | 5.31E-01 | 807553 | 0.000287896 | 232.5575636 |
| rs10235664 |  |  | T | C | 0.027 | 0.0049 | 4.68E-08 | 7.47E-01 | 807553 | 0.000252652 | 204.0811272 |
| rs12631196 |  |  | A | G | 0.0241 | 0.0044 | 3.28E-08 | 4.23E-01 | 807553 | 0.000281355 | 227.2721644 |
| rs150346963 |  |  | T | C | 0.0283 | 0.0044 | 1.16E-10 | 4.12E-01 | 807553 | 0.000281355 | 227.2721644 |
| rs3807865 |  |  | A | G | 0.031 | 0.0044 | 1.09E-12 | 4.11E-01 | 807553 | 0.000281355 | 227.2721644 |
| rs2568958 |  |  | A | G | 0.0382 | 0.0044 | 2.9E-18 | 6.04E-01 | 807553 | 0.000281355 | 227.2721644 |
| rs2247523 |  |  | C | G | -0.0243 | 0.0043 | 1.71E-08 | 5.30E-01 | 807553 | 0.000287896 | 232.5575636 |
| rs7241572 |  |  | A | G | 0.0323 | 0.0054 | 2.43E-09 | 2.05E-01 | 807553 | 0.000229264 | 185.1847266 |
| rs508502 |  |  | T | C | -0.0264 | 0.0048 | 3.56E-08 | 2.99E-01 | 807553 | 0.000257914 | 208.3328174 |
| rs9831648 |  |  | T | G | -0.0292 | 0.0052 | 1.59E-08 | 7.74E-01 | 807553 | 0.00023808 | 192.307216 |
| rs198457 |  |  | T | C | -0.0315 | 0.0056 | 1.90E-08 | 1.89E-01 | 807553 | 0.000221078 | 178.5709863 |
| rs17641524 |  |  | T | C | -0.03 | 0.0053 | 1.50E-08 | 2.10E-01 | 807553 | 0.000233589 | 188.678778 |
| rs1950829 |  |  | A | G | 0.0297 | 0.0043 | 4.74E-12 | 4.83E-01 | 807553 | 0.000287896 | 232.5575636 |
| rs7725715 |  |  | A | G | 0.029 | 0.0043 | 1.61E-11 | 5.34E-01 | 807553 | 0.000287896 | 232.5575636 |
| rs12919291 |  |  | C | G | 0.0327 | 0.0055 | 3.09E-09 | 1.88E-01 | 807553 | 0.000225096 | 181.8177315 |
| rs9831249 |  |  | A | G | -0.0247 | 0.0044 | 1.41E-08 | 5.83E-01 | 807553 | 0.000281355 | 227.2721644 |
| rs4730387 |  |  | A | T | 0.0238 | 0.0043 | 4.12E-08 | 4.66E-01 | 807553 | 0.000287896 | 232.5575636 |
| rs2214123 |  |  | A | G | 0.0261 | 0.0045 | 8.56E-09 | 3.53E-01 | 807553 | 0.000275104 | 222.2216719 |
| rs1021363 |  |  | A | G | 0.03 | 0.0045 | 2.29E-11 | 3.57E-01 | 807553 | 0.000275104 | 222.2216719 |
| rs1931388 |  |  | A | G | 0.0295 | 0.0044 | 1.68E-11 | 5.96E-01 | 807553 | 0.000281355 | 227.2721644 |
| rs62535714 |  |  | A | G | 0.0339 | 0.0058 | 4.69E-09 | 1.64E-01 | 807553 | 0.000213456 | 172.4133661 |
| rs61914045 |  |  | A | G | 0.0309 | 0.0054 | 7.96E-09 | 2.03E-01 | 807553 | 0.000229264 | 185.1847266 |
| rs2418449 |  |  | T | C | 0.0281 | 0.0048 | 4.25E-09 | 7.19E-01 | 807553 | 0.000257914 | 208.3328174 |
| rs4141983 |  |  | T | C | 0.0264 | 0.0046 | 9.69E-09 | 6.74E-01 | 807553 | 0.000269125 | 217.390766 |
| rs10913112 |  |  | T | C | -0.0262 | 0.0045 | 4.53E-09 | 3.78E-01 | 807553 | 0.000275104 | 222.2216719 |
| rs6656912 |  |  | T | C | -0.0252 | 0.0043 | 6.5E-09 | 4.27E-01 | 807553 | 0.000287896 | 232.5575636 |
| rs30266 |  |  | A | G | 0.0366 | 0.0046 | 1.43E-15 | 3.27E-01 | 807553 | 0.000269125 | 217.390766 |
| rs1367635 |  |  | T | C | -0.0253 | 0.0043 | 4.35E-09 | 4.85E-01 | 807553 | 0.000287896 | 232.5575636 |
| rs76954012 |  |  | A | T | 0.0412 | 0.0074 | 2.41E-08 | 9.31E-02 | 807553 | 0.000167311 | 135.1348005 |
| rs9364755 |  |  | A | G | -0.0283 | 0.0051 | 3.49E-08 | 7.74E-01 | 807553 | 0.000242747 | 196.0779458 |
| rs354155 |  |  | C | G | -0.0449 | 0.0075 | 1.75E-09 | 9.23E-02 | 807553 | 0.000165081 | 133.3330031 |
| rs10501696 |  |  | A | G | 0.0295 | 0.0044 | 2.89E-11 | 5.05E-01 | 807553 | 0.000281355 | 227.2721644 |
| rs9536381 |  |  | T | C | 0.0255 | 0.0046 | 2.62E-08 | 3.26E-01 | 807553 | 0.000269125 | 217.390766 |
| rs72948506 |  |  | A | G | 0.0265 | 0.0047 | 1.72E-08 | 2.98E-01 | 807553 | 0.000263401 | 212.7654305 |
| rs9529218 |  |  | T | C | -0.034 | 0.0054 | 2.23E-10 | 2.03E-01 | 807553 | 0.000229264 | 185.1847266 |
| rs150186873 |  |  | A | C | -0.0704 | 0.012 | 4.51E-09 | 9.67E-01 | 807553 | 0.000103182 | 83.33312695 |
| rs59082935 |  |  | T | C | 0.0363 | 0.0066 | 3.07E-08 | 1.34E-01 | 807553 | 0.000187587 | 151.5147763 |
| rs28541419 |  |  | C | G | 0.0292 | 0.0052 | 1.76E-08 | 7.69E-01 | 807553 | 0.00023808 | 192.307216 |
| rs12967143 |  |  | C | G | -0.0345 | 0.0047 | 2.53E-13 | 7.01E-01 | 807553 | 0.000263401 | 212.7654305 |
| rs754287 |  |  | A | T | -0.0289 | 0.0045 | 1.31E-10 | 3.66E-01 | 807553 | 0.000275104 | 222.2216719 |
| **SNP** | **chr** | **pos** | **effect_allele** | **other_allele** | **BETA** | **SE** | **P-value** | **EAF** | **samplesize** | **R2** | **F-statistic** |
| **Bipolar Disorder** | | | | | | | | | | | |
| rs174592 | 11 | 61618608 | A | G | -0.076881044 | 0.0141 | 3.66E-08 | 6.18E-01 | 51710 | 0.001369655 | 70.91924275 |
| rs73496688 | 11 | 79156748 | A | T | 0.104360015 | 0.019 | 1.05E-08 | 1.57E-01 | 51710 | 0.001016787 | 52.6295433 |
| rs10744560 | 12 | 2387099 | T | C | 0.086177696 | 0.014 | 2.92E-09 | 3.52E-01 | 51710 | 0.001379425 | 71.42580877 |
| rs71395455 | 15 | 85153804 | A | G | 0.086177696 | 0.0146 | 1.93E-08 | 6.97E-01 | 51710 | 0.001322811 | 68.49050156 |
| rs884301 | 17 | 53367464 | T | C | 0.076961041 | 0.0138 | 5.8E-09 | 3.92E-01 | 51710 | 0.001399388 | 72.46096542 |
| rs111444407 | 19 | 19358207 | T | C | 0.113328685 | 0.0184 | 2.4E-10 | 1.65E-01 | 51710 | 0.001049908 | 54.34572406 |
| rs2314398 | 2 | 97413488 | C | G | 0.086177696 | 0.0144 | 5.92E-09 | 6.95E-01 | 51710 | 0.001341159 | 69.44175853 |
| rs138321 | 22 | 41209304 | A | G | 0.076961041 | 0.0135 | 4.69E-09 | 5.22E-01 | 51710 | 0.001430441 | 74.07120909 |
| rs9834970 | 3 | 36856030 | T | C | -0.100925919 | 0.0134 | 5.53E-14 | 4.83E-01 | 51710 | 0.001441101 | 74.62397931 |
| rs2302417 | 3 | 52814256 | A | T | -0.079043207 | 0.0136 | 4.93E-09 | 4.72E-01 | 51710 | 0.001419938 | 73.52656785 |
| rs11724116 | 4 | 162294038 | T | C | -0.104250021 | 0.0188 | 3.27E-08 | 1.48E-01 | 51710 | 0.001027593 | 53.18943206 |
| rs329319 | 5 | 133906609 | A | G | 0.076961041 | 0.0139 | 1.54E-08 | 4.44E-01 | 51710 | 0.001389335 | 71.93966351 |
| rs55648125 | 6 | 50816718 | A | G | -0.116533816 | 0.0215 | 4.92E-08 | 8.85E-01 | 51710 | 0.000898662 | 46.50982897 |
| rs10455979 | 6 | 166995260 | C | G | -0.074723546 | 0.0137 | 4.60E-08 | 5.15E-01 | 51710 | 0.001409588 | 72.98987757 |
| rs17150022 | 7 | 24771777 | T | C | -0.113168698 | 0.0204 | 2.70E-08 | 8.72E-01 | 51710 | 0.000947074 | 49.0177119 |
| rs13231398 | 7 | 110197412 | C | G | -0.121038328 | 0.0219 | 3.36E-08 | 1.01E-01 | 51710 | 0.000882263 | 45.66033437 |
| SNP single nucleotide polymorphism, Chr chromosome, Pos Position, se standard error, eaf effect allele frequency. | | | | | | | | | | | |
