# Supplementary material for: The causal relationship between severe mental illness and risk of lung carcinoma
Source: Medicine (Baltimore). 2024 Mar 15;103(11):e37355. doi: 10.1097/MD.0000000000037355 (PMC10939700; doi:10.1097/MD.0000000000037355)
Supplement: Supplementary file 3 [file medi-103-e37355-s003.docx]

| **Table S3 Summary information extraction for SNPs associated with schizophrenia, MDD, and BD in lung carcinoma outcomes** | | | | | | |
| --- | --- | --- | --- | --- | --- | --- |
|  |  |  |  |  |  |  |
| **SNP** | **Effect allele** | **Other allele** | **Beta** | **EAF** | **SE** | **P-value** |
| **schizophrenia** |  |  |  |  |  |  |
| rs1000237 | T | A | -0.073205303 | 0.627 | 0.0089 | 2.8E-16 |
| rs10035564 | A | G | -0.066802415 | 0.65 | 0.0092 | 4.38E-13 |
| rs10086619 | A | G | -0.072205168 | 0.831 | 0.0116 | 4.97E-10 |
| rs10108980 | C | T | -0.062801364 | 0.785 | 0.0106 | 2.73E-09 |
| rs10117 | G | A | 0.054999418 | 0.613 | 0.0088 | 4.66E-10 |
| rs10861176 | G | A | -0.055502139 | 0.257 | 0.0098 | 1.59E-08 |
| rs10876446 | G | C | -0.054002223 | 0.674 | 0.0094 | 1.03E-08 |
| rs11027839 | A | C | -0.051503843 | 0.488 | 0.0086 | 2.4E-09 |
| rs11136325 | G | A | 0.053796658 | 0.436 | 0.0091 | 3.05E-09 |
| rs11165867 | C | T | -0.074303376 | 0.829 | 0.0116 | 1.3E-10 |
| rs11191580 | T | C | 0.131703473 | 0.92 | 0.0155 | 1.77E-17 |
| rs11223774 | A | G | 0.052497569 | 0.301 | 0.0094 | 2.74E-08 |
| rs113264400 | T | C | -0.112295619 | 0.948 | 0.0202 | 2.87E-08 |
| rs11534045 | G | A | 0.062796379 | 0.686 | 0.0093 | 1.4E-11 |
| rs11587347 | C | G | -0.103894923 | 0.895 | 0.0147 | 1.53E-12 |
| rs11664298 | G | A | -0.077399537 | 0.794 | 0.0108 | 8.94E-13 |
| rs11693094 | C | T | 0.054402954 | 0.556 | 0.0087 | 4.29E-10 |
| rs117178087 | C | T | 0.096400494 | 0.939 | 0.0177 | 4.89E-08 |
| rs11740474 | A | T | -0.053696179 | 0.575 | 0.0088 | 1.13E-09 |
| rs1198588 | A | T | -0.102597669 | 0.194 | 0.0108 | 1.73E-21 |
| rs12129573 | C | A | -0.077799392 | 0.616 | 0.0089 | 2.28E-18 |
| rs12138231 | T | A | -0.066994869 | 0.171 | 0.0116 | 7.99E-09 |
| rs12151767 | G | A | 0.061104507 | 0.524 | 0.0086 | 1.31E-12 |
| rs12285419 | C | A | -0.084904507 | 0.8 | 0.011 | 1.05E-14 |
| rs12293670 | A | G | 0.070495742 | 0.678 | 0.0092 | 1.56E-14 |
| rs12303743 | G | C | -0.087498768 | 0.898 | 0.0145 | 1.59E-09 |
| rs12652777 | T | C | 0.048799688 | 0.489 | 0.0086 | 1.52E-08 |
| rs12712510 | T | C | 0.057400607 | 0.488 | 0.0087 | 5.14E-11 |
| rs12771371 | G | A | 0.052402679 | 0.697 | 0.0093 | 1.94E-08 |
| rs12833624 | C | T | -0.050199156 | 0.646 | 0.009 | 2.77E-08 |
| rs12877581 | G | C | -0.059601395 | 0.716 | 0.0099 | 1.8E-09 |
| rs12883788 | C | T | -0.061301101 | 0.527 | 0.0087 | 1.86E-12 |
| rs13016542 | T | C | 0.088303875 | 0.876 | 0.0129 | 8.28E-12 |
| rs13233308 | C | T | 0.048704446 | 0.526 | 0.0086 | 1.75E-08 |
| rs132582 | C | T | 0.050997251 | 0.471 | 0.0086 | 3.26E-09 |
| rs1427633 | G | C | 0.048304332 | 0.419 | 0.0088 | 0.000000041 |
| rs1430894 | C | T | -0.053295297 | 0.506 | 0.0086 | 6.15E-10 |
| rs145071536 | T | C | -0.085100477 | 0.799 | 0.012 | 1.62E-12 |
| rs1451488 | A | G | -0.070894679 | 0.432 | 0.0087 | 4.47E-16 |
| rs149165 | T | G | 0.048199514 | 0.569 | 0.0087 | 3.01E-08 |
| rs1540840 | G | C | 0.055699572 | 0.542 | 0.0093 | 2.21E-09 |
| rs1593304 | A | G | -0.064101283 | 0.193 | 0.0111 | 7.45E-09 |
| rs167924 | A | G | -0.050199156 | 0.359 | 0.009 | 2.34E-08 |
| rs16851048 | T | C | -0.074497279 | 0.792 | 0.0107 | 4.15E-12 |
| rs16867571 | A | G | 0.065703467 | 0.778 | 0.0104 | 2.68E-10 |
| rs17016552 | C | G | 0.051700212 | 0.661 | 0.0091 | 0.000000012 |
| rs17731 | G | A | -0.052399169 | 0.62 | 0.0089 | 4.37E-09 |
| rs187557 | C | T | 0.066695568 | 0.163 | 0.0119 | 2.03E-08 |
| rs1881046 | G | T | 0.05070262 | 0.67 | 0.0092 | 3.39E-08 |
| rs1892346 | T | A | -0.048402739 | 0.428 | 0.0088 | 3.56E-08 |
| rs1901512 | T | C | 0.058400975 | 0.318 | 0.0094 | 5.72E-10 |
| rs1914399 | C | G | 0.049104401 | 0.489 | 0.0087 | 0.000000014 |
| rs1915019 | A | G | 0.057098412 | 0.265 | 0.0098 | 6.57E-09 |
| rs1953205 | T | A | -0.049904785 | 0.506 | 0.0089 | 2.22E-08 |
| rs2053079 | A | G | -0.059898635 | 0.754 | 0.0101 | 3.01E-09 |
| rs2078266 | A | G | 0.069600687 | 0.178 | 0.0126 | 2.94E-08 |
| rs215412 | G | A | -0.057703268 | 0.661 | 0.0091 | 2.69E-10 |
| rs217336 | C | A | 0.050303305 | 0.583 | 0.0087 | 8.05E-09 |
| rs2238057 | T | G | -0.083501181 | 0.568 | 0.0087 | 8.5E-22 |
| rs2252074 | T | G | -0.068503705 | 0.59 | 0.0088 | 6.19E-15 |
| rs2332700 | C | G | 0.075098196 | 0.258 | 0.0099 | 3.88E-14 |
| rs2333321 | A | G | 0.071203759 | 0.218 | 0.0105 | 1.25E-11 |
| rs2381411 | T | C | -0.050398958 | 0.586 | 0.0088 | 1.25E-08 |
| rs2455415 | C | T | -0.049494912 | 0.577 | 0.0088 | 1.69E-08 |
| rs2710323 | T | C | 0.078404443 | 0.531 | 0.0086 | 1.23E-19 |
| rs2815731 | C | A | 0.060003252 | 0.66 | 0.0091 | 4.39E-11 |
| rs2999392 | C | T | -0.051798685 | 0.299 | 0.0094 | 3.05E-08 |
| rs308697 | C | A | 0.050103587 | 0.574 | 0.0087 | 8.83E-09 |
| rs35351411 | A | C | -0.063504389 | 0.439 | 0.0087 | 2.21E-13 |
| rs35734242 | T | C | -0.050703994 | 0.562 | 0.0089 | 1.37E-08 |
| rs3739118 | G | A | 0.057003957 | 0.719 | 0.0095 | 2.36E-09 |
| rs3770754 | C | G | 0.052896009 | 0.645 | 0.0091 | 5.35E-09 |
| rs3791710 | T | C | 0.060003252 | 0.804 | 0.0108 | 3.02E-08 |
| rs3795310 | C | T | 0.050997251 | 0.543 | 0.0087 | 5.75E-09 |
| rs3802924 | A | C | 0.073603559 | 0.805 | 0.0108 | 9.58E-12 |
| rs3824451 | T | C | -0.065595079 | 0.838 | 0.0118 | 2.54E-08 |
| rs4575535 | A | G | -0.055798163 | 0.281 | 0.0096 | 5.77E-09 |
| rs4653164 | C | T | -0.051103839 | 0.323 | 0.0092 | 3.08E-08 |
| rs4700418 | C | G | -0.070197168 | 0.489 | 0.0087 | 5.37E-16 |
| rs4766428 | C | T | -0.075003758 | 0.539 | 0.0089 | 3.93E-17 |
| rs4779050 | T | G | 0.057995286 | 0.381 | 0.0089 | 7.27E-11 |
| rs4812325 | G | A | -0.071904248 | 0.37 | 0.0089 | 8.96E-16 |
| rs4921741 | A | G | -0.055999086 | 0.729 | 0.0098 | 1.21E-08 |
| rs498591 | A | T | -0.072495427 | 0.847 | 0.0121 | 2.11E-09 |
| rs505061 | C | A | -0.053495718 | 0.495 | 0.0086 | 5.8E-10 |
| rs56205728 | G | A | -0.0630037 | 0.7 | 0.0097 | 1.01E-10 |
| rs56335113 | A | G | 0.064701008 | 0.316 | 0.0094 | 6.02E-12 |
| rs57433322 | C | G | 0.083099569 | 0.885 | 0.0139 | 1.99E-09 |
| rs5751191 | T | C | -0.065595079 | 0.484 | 0.0086 | 3E-14 |
| rs58120505 | T | C | 0.089603016 | 0.602 | 0.0088 | 2.24E-24 |
| rs6125656 | G | A | -0.064495855 | 0.809 | 0.0111 | 6.29E-09 |
| rs61857878 | A | T | 0.060097424 | 0.76 | 0.0102 | 4.44E-09 |
| rs61937595 | C | T | 0.130098005 | 0.917 | 0.0162 | 1.15E-15 |
| rs62018952 | T | C | -0.058402717 | 0.264 | 0.0097 | 1.94E-09 |
| rs62183855 | A | C | 0.066096682 | 0.814 | 0.0111 | 2.66E-09 |
| rs634940 | G | T | -0.066396246 | 0.737 | 0.0099 | 1.78E-11 |
| rs6482437 | A | C | -0.098903629 | 0.0994 | 0.0142 | 3.33E-12 |
| rs6520064 | A | G | -0.058498134 | 0.787 | 0.0106 | 3.58E-08 |
| rs6538539 | G | T | 0.056796126 | 0.462 | 0.0086 | 4.43E-11 |
| rs6546857 | A | G | -0.060397773 | 0.757 | 0.0102 | 2.74E-09 |
| rs6549963 | T | C | 0.048304332 | 0.596 | 0.0088 | 4.31E-08 |
| rs6673880 | A | G | -0.062301026 | 0.492 | 0.0091 | 7.2E-12 |
| rs6798742 | A | G | -0.061099109 | 0.68 | 0.0093 | 4.57E-11 |
| rs6943762 | T | C | 0.105098481 | 0.883 | 0.0132 | 1.57E-15 |
| rs6974218 | A | C | 0.054895299 | 0.634 | 0.0089 | 6.8E-10 |
| rs7112616 | T | C | 0.05220338 | 0.515 | 0.0086 | 1.52E-09 |
| rs713692 | G | A | -0.056602098 | 0.299 | 0.0095 | 2.67E-09 |
| rs7251 | C | G | 0.064100925 | 0.682 | 0.0094 | 8.29E-12 |
| rs72802868 | G | T | 0.069199517 | 0.724 | 0.0096 | 4.55E-13 |
| rs728055 | T | A | 0.067396932 | 0.658 | 0.009 | 8.85E-14 |
| rs72943392 | G | C | -0.053495718 | 0.707 | 0.0096 | 2.39E-08 |
| rs72986630 | C | T | -0.112295619 | 0.926 | 0.0179 | 3.59E-10 |
| rs73292401 | T | A | -0.06760455 | 0.799 | 0.0109 | 5.48E-10 |
| rs7515363 | C | T | 0.053502852 | 0.389 | 0.0089 | 1.84E-09 |
| rs7575796 | A | G | 0.096300598 | 0.917 | 0.0172 | 2.07E-08 |
| rs7634476 | A | G | -0.057703268 | 0.397 | 0.0088 | 5.46E-11 |
| rs7647398 | C | T | 0.077497934 | 0.811 | 0.0109 | 1.07E-12 |
| rs76838079 | C | T | -0.078004786 | 0.849 | 0.0138 | 1.53E-08 |
| rs778371 | A | G | -0.080602865 | 0.699 | 0.0095 | 1.5E-17 |
| rs7798283 | T | G | 0.074002967 | 0.875 | 0.0134 | 3.49E-08 |
| rs7830315 | T | C | -0.04780465 | 0.478 | 0.0086 | 3.08E-08 |
| rs79210963 | T | C | -0.085601462 | 0.884 | 0.0137 | 4.14E-10 |
| rs79445414 | T | C | -0.123400031 | 0.956 | 0.0222 | 0.000000028 |
| rs9304548 | C | A | 0.056701643 | 0.261 | 0.01 | 1.59E-08 |
| rs9318627 | A | C | 0.061198575 | 0.613 | 0.0088 | 4.35E-12 |
| rs9461916 | T | C | -0.053295297 | 0.388 | 0.0088 | 1.64E-09 |
| rs9636107 | A | G | -0.069896851 | 0.506 | 0.0086 | 5.12E-16 |
| rs9687282 | T | G | -0.05259941 | 0.65 | 0.0091 | 7.33E-09 |
| rs9876421 | C | T | -0.06250326 | 0.645 | 0.0092 | 9.19E-12 |
| **MDD** |  |  |  |  |  |  |
| rs1021363 | A | G | 0.03 | 0.3566 | 0.0045 | 2.29E-11 |
| rs10235664 | T | C | 0.027 | 0.7471 | 0.0049 | 4.68E-08 |
| rs10501696 | A | G | 0.0295 | 0.5052 | 0.0044 | 2.89E-11 |
| rs10913112 | T | C | -0.0262 | 0.378 | 0.0045 | 4.53E-09 |
| rs12919291 | C | G | 0.0327 | 0.1884 | 0.0055 | 3.09E-09 |
| rs12967143 | C | G | -0.0345 | 0.7012 | 0.0047 | 2.53E-13 |
| rs13037326 | T | C | 0.031 | 0.2597 | 0.0049 | 2.4E-10 |
| rs1367635 | T | C | -0.0253 | 0.4852 | 0.0043 | 4.35E-09 |
| rs150186873 | A | C | -0.0704 | 0.9673 | 0.012 | 4.51E-09 |
| rs150346963 | T | C | 0.0283 | 0.4118 | 0.0044 | 1.16E-10 |
| rs17641524 | T | C | -0.03 | 0.2101 | 0.0053 | 0.000000015 |
| rs1931388 | A | G | 0.0295 | 0.5958 | 0.0044 | 1.68E-11 |
| rs1950829 | A | G | 0.0297 | 0.4827 | 0.0043 | 4.74E-12 |
| rs2111592 | A | G | 0.0263 | 0.3141 | 0.0046 | 1.35E-08 |
| rs2214123 | A | G | 0.0261 | 0.3534 | 0.0045 | 8.56E-09 |
| rs2418449 | T | C | 0.0281 | 0.719 | 0.0048 | 4.25E-09 |
| rs2568958 | A | G | 0.0382 | 0.6042 | 0.0044 | 2.9E-18 |
| rs28541419 | C | G | 0.0292 | 0.7692 | 0.0052 | 1.76E-08 |
| rs30266 | A | G | 0.0366 | 0.3271 | 0.0046 | 1.43E-15 |
| rs3099439 | T | C | -0.0241 | 0.5351 | 0.0043 | 2.78E-08 |
| rs354155 | C | G | -0.0449 | 0.0923 | 0.0075 | 1.75E-09 |
| rs3807865 | A | G | 0.031 | 0.4105 | 0.0044 | 1.09E-12 |
| rs4141983 | T | C | 0.0264 | 0.674 | 0.0046 | 9.69E-09 |
| rs508502 | T | C | -0.0264 | 0.2992 | 0.0048 | 3.56E-08 |
| rs59082935 | T | C | 0.0363 | 0.1342 | 0.0066 | 3.07E-08 |
| rs59283172 | A | G | -0.039 | 0.1081 | 0.007 | 2.41E-08 |
| rs62535714 | A | G | 0.0339 | 0.1639 | 0.0058 | 4.69E-09 |
| rs6656912 | T | C | -0.0252 | 0.4273 | 0.0043 | 6.5E-09 |
| rs7152906 | T | C | -0.0258 | 0.4804 | 0.0043 | 1.87E-09 |
| rs7241572 | A | G | 0.0323 | 0.2047 | 0.0054 | 2.43E-09 |
| rs72948506 | A | G | 0.0265 | 0.2975 | 0.0047 | 1.72E-08 |
| rs754287 | A | T | -0.0289 | 0.3664 | 0.0045 | 1.31E-10 |
| rs76954012 | A | T | 0.0412 | 0.0931 | 0.0074 | 2.41E-08 |
| rs7725715 | A | G | 0.029 | 0.5343 | 0.0043 | 1.61E-11 |
| rs9364755 | A | G | -0.0283 | 0.7738 | 0.0051 | 3.49E-08 |
| rs9529218 | T | C | -0.034 | 0.2031 | 0.0054 | 2.23E-10 |
| rs9536381 | T | C | 0.0255 | 0.3259 | 0.0046 | 2.62E-08 |
| rs9831249 | A | G | -0.0247 | 0.5826 | 0.0044 | 1.41E-08 |
| rs9831648 | T | G | -0.0292 | 0.7739 | 0.0052 | 1.59E-08 |
| **BD** |  |  |  |  |  |  |
| rs10455979 | C | G | -0.074723546 | 0.515 | 0.0137 | 0.000000046 |
| rs10744560 | T | C | 0.086177696 | 0.352 | 0.014 | 2.92E-09 |
| rs111444407 | T | C | 0.113328685 | 0.165 | 0.0184 | 2.4E-10 |
| rs11724116 | T | C | -0.104250021 | 0.148 | 0.0188 | 3.27E-08 |
| rs13231398 | C | G | -0.121038328 | 0.101 | 0.0219 | 3.36E-08 |
| rs138321 | A | G | 0.076961041 | 0.522 | 0.0135 | 4.69E-09 |
| rs17150022 | T | C | -0.113168698 | 0.872 | 0.0204 | 0.000000027 |
| rs174592 | A | G | -0.076881044 | 0.618 | 0.0141 | 3.66E-08 |
| rs2302417 | A | T | -0.079043207 | 0.472 | 0.0136 | 4.93E-09 |
| rs2314398 | C | G | 0.086177696 | 0.695 | 0.0144 | 5.92E-09 |
| rs329319 | A | G | 0.076961041 | 0.444 | 0.0139 | 1.54E-08 |
| rs55648125 | A | G | -0.116533816 | 0.885 | 0.0215 | 4.92E-08 |
| rs71395455 | A | G | 0.086177696 | 0.697 | 0.0146 | 1.93E-08 |
| rs73496688 | A | T | 0.104360015 | 0.157 | 0.019 | 1.05E-08 |
| rs884301 | T | C | 0.076961041 | 0.392 | 0.0138 | 5.8E-09 |
| rs9834970 | T | C | -0.100925919 | 0.483 | 0.0134 | 5.53E-14 |
| MDD major depressive disorder, BD bipolar disorder, SNP single nucleotide polymorphism, SE standard error, EAF effect allele frequency. | | | | | | |
|  |  |  |  |  |  |  |
