# Supplementary material for: The causal relationship between severe mental illness and risk of lung carcinoma
Source: Medicine (Baltimore). 2024 Mar 15;103(11):e37355. doi: 10.1097/MD.0000000000037355 (PMC10939700; doi:10.1097/MD.0000000000037355)
Supplement: Supplementary file 4 [file medi-103-e37355-s004.docx]

| **Table S4 Initial Mendelian randomization results of the causal association between schizophrenia, major depression and bipolar disorder** | | | | | |
| --- | --- | --- | --- | --- | --- |
|  |  |  |  |  |  |
| Exposure | SNPs, n | Methods | OR | 95% CI | P-value |
| Schizophrenia | 129 | Inverse variance weighted | 1.06 | 1.02, 1.11 | 3.48E-03 |
|  | 129 | Weighted median | 1.08 | 1.02, 1.14 | 4.29E-03 |
|  | 129 | MR−Egger | 1.00 | 0.82, 1.22 | 0.99 |
|  | 129 | MR−PRESSO | 1.06 | 1.02, 1.11 | 4.12E-03 |
| Major Depression (Initial MR) | 40 | Inverse variance weighted | 1.14 | 0.94, 1.39 | 0.20 |
|  | 40 | Weighted median | 1.20 | 0.97, 1.48 | 0.09 |
|  | 40 | MR−Egger | 1.02 | 0.30, 3.46 | 0.97 |
|  | 40 | MR−PRESSO | 1.15 | 0.95, 1.39 | 0.15 |
| Bipolar Disorder | 16 | Inverse variance weighted (fixed) | 1.07 | 0.99, 1.15 | 0.09 |
|  | 16 | Weighted median | 1.05 | 0.95, 1.17 | 0.33 |
|  | 16 | MR−Egger | 1.59 | 0.98, 2.59 | 0.08 |
|  | 16 | MR−PRESSO | 1.07 | 0.98, 1.16 | 0.14 |
